# Loss of RPA1 Impairs Peripheral T Cell Homeostasis and Exacerbates Inflammatory Damage through Triggering T Cell Necroptosis

**DOI:** 10.1002/advs.202206344

**Published:** 2023-01-31

**Authors:** Jia Song, Xin Zhang, Yue Yin, Mengfan Guo, Xuyang Zhao, Likun Wang, Caixia Ren, Yuxin Yin, Xuehui Zhang, Xuliang Deng, Dan Lu

**Affiliations:** ^1^ Department of Geriatric Dentistry Department of Dental Materials & Dental Medical Devices Testing Center National Engineering Research Center of Oral Biomaterials and Digital Medical Devices NMPA Key Laboratory for Dental Materials Beijing Laboratory of Biomedical Materials & Beijing Key Laboratory of Digital Stomatology Peking University School and Hospital of Stomatology Beijing 100081 P. R. China; ^2^ Institute of Systems Biomedicine Department of Pathology School of Basic Medical Sciences Peking University Health Science Center Beijing 100191 P. R. China; ^3^ Department of Human Anatomy Histology and Embryology Peking University Health Science Center Beijing 100191 P. R. China

**Keywords:** inflammatory damage, necroptosis, replication protein A1 (RPA1), T cell homeostasis, T cell receptors (TCR) repertoire

## Abstract

The peripheral T cell pool is maintained at dynamic homeostasis through fine‐tuning of thymic output and self‐renewal of naïve T cells. Lymphopenia or reduced lymphocyte number is implicated in autoimmune diseases, yet little is known about the homeostatic mechanisms. Here, it is reported that the replication protein A1 (RPA1) plays a critical role in T cell homeostasis. Utilizing T cell‐specific *Rpa1*‐deficient (*Rpa1^fl/fl^ Cd4‐cre*) mice, loss of *Rpa1* results in lymphopenia through restraining peripheral T cell population and limiting TCR repertoire diversity. Moreover, *Rpa1^fl/fl^ Cd4‐cre* mice exhibit increased susceptibility to inflammatory diseases, including colitis and hepatitis. Clinical analysis reveals that the expression of RPA1 is reduced in patients with ulcerative colitis or other autoinflammatory diseases. Mechanistically, depletion of RPA1 activates ZBP1‐RIPK3 signaling through triggering the genomic DNA leakage into cytosol, consequently resulting in T cell necroptosis. This necroptotic T cell death induced by RPA1 deficiency allows the release of damage‐associated molecular patterns (DAMPs), which in turn recruits leukocytes and exacerbates inflammatory response. Reciprocally, chemical or genetic inhibition of necroptosis signaling can ameliorate the *Rpa1* deficiency‐induced inflammatory damage. The studies thus uncover the importance of RPA1‐ZBP1‐RIPK3 axis in T cell homeostasis and provide a promising strategy for autoinflammatory disease treatment.

## Graphical abstract

Lymphopenia or reduced lymphocyte number is implicated in autoimmune diseases, yet little is known about the regulatory mechanism. Here, it is reported RPA1‐ZBP1 axis is crucial for T cell homeostasis. Conversely, depletion of T cell‐intrinsic RPA1 results in lymphopenia and increases susceptibility to autoimmune disease through inducing T cell necroptosis.

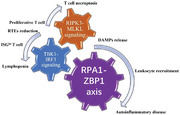

## 1 Introduction

T cells begin to develop in thymus and subsequently emigrate to peripheral tissues to establish the mature T cell pool.^[^ ^1^ ^]^ Under physiological condition, the T cell system is in constant turnover and maintained dynamic homeostasis.^[^ ^2^ ^]^ It has been estimated that 20–50% of T lymphocytes have a life span of several days to a few weeks.^[^ ^3^ ^]^ Two major mechanisms including generation of new thymic emigrants and peripheral self‐renewal, contribute to the lymphocyte replenishment.^[^ ^4^, ^5^ ^]^

Lymphopenia or reduced lymphocyte number can trigger lymphopenia‐induced proliferation (LIP) to sustain a relative constant amount of peripheral T lymphocytes but also maintain the diversity of T cell repertoire.^[^ ^6^ ^]^ In contrast to the minimal proliferative rate of naïve T cell in a full lymphocyte compartment under physiological circumstance, which has been referred to as “basal proliferation” or “spontaneous proliferation,” the LIP describes space‐driven expansion of T cells as a robust compensatory mechanism for restoration of their numbers under conditions of lymphopenia, which is also correlated with autoinflammatory response.^[^ ^7^ ^]^ Elucidation of mechanisms by which T cell homeostasis is regulated are therefore critical for the treatment of autoinflammatory diseases. In addition to the critical role of IL‐7 and its receptor IL‐7R in T cell survival, clonal expansion also contributes to the maintenance of T cell population.^[^ ^8^, ^9^ ^]^ However, robust cell proliferation can trigger replication stress including DNA lesion, unusual secondary DNA structure and replication‐fork obstacles, which induce cell senescence, apoptosis and necroptosis.^[^ ^10^, ^11^, ^12^ ^]^ It is conceivable that a series of factors participate in the process to initiate DNA repair, restrict cell death and maintain a relatively constant T cell population. Although activation of IL‐7‐IL‐7R signaling blocks the biological program of apoptosis, relatively little is known about other nuclear factors that modulate the replication stress during T cell expansion.

Replication protein A1 (RPA1) is conserved in eukaryotes and plays a key role in DNA replication, recombination and repair.^[^ ^13^ ^]^ As a single‐stranded DNA (ssDNA) binding protein, RPA1 sensors accumulative ssDNA induced by lagging strand DNA synthesis, which in turn limits fork fragility and activates Ataxia telangiectasia and Rad3‐related protein (ATR)‐mediated signaling, consequently contributing to the maintenance of genomic integrity.^[^ ^14^ ^]^ In addition to ssDNA, RPA1 can also bind to the free DNA fragments generated as DNA repair intermediates during nucleolytic processing at stalled replication forks.^[^ ^15^ ^]^ Through prevention of free DNA fragments leakage into cytoplasm, RPA1 restricts the activation of pattern‐recognition receptors including cyclic GMP‐AMP (cGAS)‐cyclic GMP‐AMP receptor stimulator of interferon genes (STING), Z‐DNA‐binding protein (ZBP1/DAI) and apoptosis‐inducing factor mitochondria‐associated 2 (AIM2).^[^ ^16^, ^17^, ^18^ ^]^ Among them, ZBP1 has been found to play an immunostimulatory role in blood lymphocytes during neuroinflammation and may be involved in the induction of necroptosis.^[^ ^19^ ^]^ However, the role of RPA1‐ZBP1 axis in T‐cell biology remains unknown.

In this study, we characterized the essential role of RPA1 in T cell homeostasis, which is downregulated in autoinflammatory disease. Using T cell‐intrinsic RPA1 deficient mice, we found that loss of RPA1 reduced peripheral T cells numbers and skewed TCR repertoire of CD8^+^ T cells. Mechanistic studies showed that deletion of RPA1 activated ZBP1 signaling by inducing genomic DNA leakage into cytosol. Through TBK1‐IRF3 and RIPK3‐MLKL signaling, RPA1 deficiency initiated necroptotic cell death on recent thymic emigrants and proliferative T cells, consequently resulting in lymphopenia and increased susceptibility to autoimmune diseases. Our study therefore identifies RPA1 as a guardian of T cell renewal and highlights the importance of targeting RPA1 in the treatment of autoinflammatory diseases.

## 2 Results

### 2.1 RPA1 is Upregulated During T Cell Expansion

**Figure 1:**
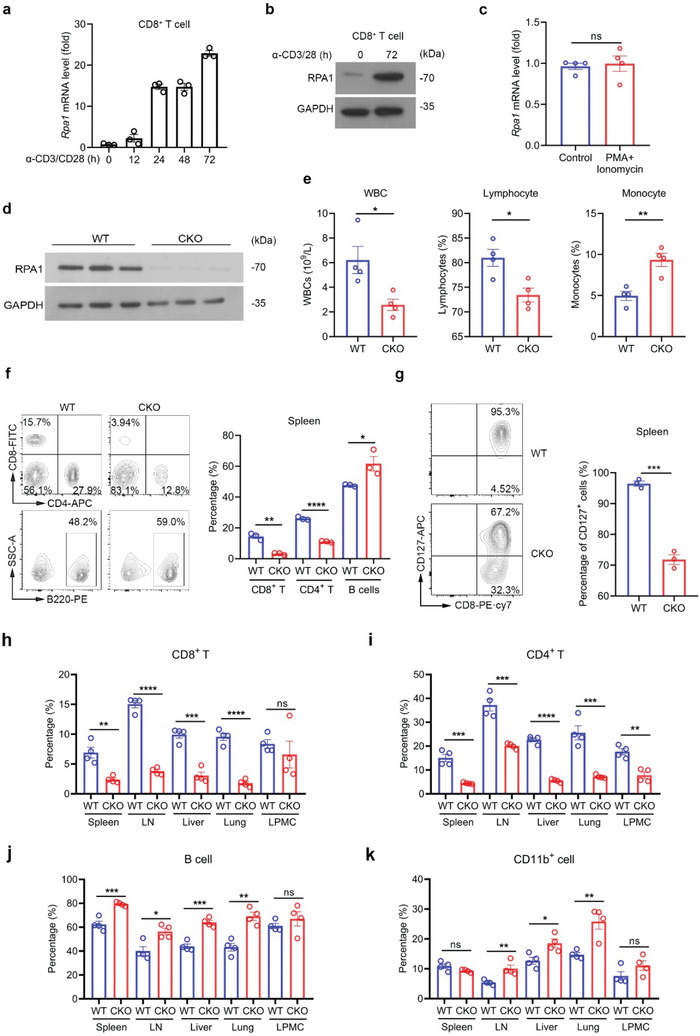
RPA1 is critical for dynamic homeostasis of peripheral T cell pool. a) Quantitative real‐time PCR (RT‐qPCR) analysis of *Rpa1* mRNA level in naïve CD8^+^ T lymphocytes derived from 6‐week‐old wild‐type (WT) mice spleen. Antibodies against CD3 (2 µg mL^−1^) and CD28 (1 µg mL^−1^) were used to activate naïve T cells (*n* = 3 biological replicates, mean ± s.e.m.). The primers used for RT‐qPCR have been deposited in Table S1 (Supporting Information). b) Immunoblot analysis of protein level of RPA1 in naïve CD8^+^ T cells derived from 6‐week‐old WT mice spleen with or without anti‐CD3/CD28 antibody treatment. c) RT‐qPCR analysis of *Rpa1* mRNA level in naïve CD8^+^ T cells derived from 6‐week‐old WT mice spleen. PMA (100 ng mL^−1^) and ionomycin (500 ng mL^−1^) were used to stimulate T cell activation (*n* = 4 biological replicates, mean ± s.e.m., ns, not significant (*P* > 0.05), two‐tailed unpaired Student's *t*‐test). The primers used for RT‐qPCR have been deposited in Table S1 (Supporting Information). d) Immunoblot analysis of protein level of RPA1 in T cells derived from 6‐week‐old WT and RPA1 conditional knockout (CKO) mice spleen (*n* = 3). e) Quantities of white blood cells (WBCs), lymphocytes and monocytes in 6‐week‐old male WT and CKO mice were analyzed by routine blood test using a HEMAVET 950FS Veterinary Multi‐species Hematology System (*n* = 4 biological replicates, mean ± s.e.m., *P < 0.05, **P = 0.0041, two‐tailed unpaired Student's *t*‐test). f) Flow cytometric analysis of the frequencies of CD4^+^ T cell, CD8^+^ T cell and B cell subsets in spleen from 6‐week‐old male WT and CKO mice (*n* = 3 mice, mean ± s.e.m., *P = 0.0409, **P = 0.0010, ****P < 0.0001, two‐tailed unpaired Student's *t*‐test). g) Flow cytometric analysis of CD127^+^ cells frequency in CD8^+^ T cells derived from 6‐week‐old male WT and CKO mice spleen (*n* = 3 mice, mean ± s.e.m., ***P = 0.0002, two‐tailed unpaired Student's *t*‐test). h–k) Flow cytometric analysis of the frequencies of CD8^+^ T cells (*n* = 4 mice, mean ± s.e.m., ns, not significant (*P* > 0.05), **P = 0.0030, ***P = 0.0002, ****P < 0.0001, two‐tailed unpaired Student's *t*‐test) h), CD4^+^ T cells (*n* = 4 mice, mean ± s.e.m., **P = 0.0018, ***P < 0.001, ****P < 0.0001, two‐tailed unpaired Student's *t*‐test) i), B cells (*n* = 4 mice, mean ± s.e.m., ns, not significant (*P* > 0.05), *P = 0.0119, **P = 0.0014, ***P < 0.001, two‐tailed unpaired Student's *t*‐test) j) and CD11b^+^ cells (*n* = 4 mice, mean ± s.e.m., ns, not significant (*P* > 0.05), *P = 0.0179, **P < 0.01, two‐tailed unpaired Student's *t*‐test) k) in spleen, lymph node (LN), liver, lung and lamina propria mononuclear cell (LPMC) from 6‐week‐old male WT and CKO mice.

T cell immunity requires robust antigen‐specific T cell proliferation to form effector T cells.^[^ ^1^ ^]^ As the initiator of DNA repair, RPA1 is highly expressed in immune system, including lymph node (LN) and spleen (Figure S1A,B, Supporting Information). To assess the status of RPA1 in T cell activation and ensued expansion, we sorted the CD4^+^ or CD8^+^ naïve T cells (CD25^−^CD44^lo^CD62L^hi^) from C57BL/6 mice by flow cytometry and activated the cells for various times with anti‐CD3 and anti‐CD28 antibodies, then analyzed the mRNA level of *Rpa1* by quantitative RT‐PCR. As shown in **Figure** **1**a and Figure S1C (Supporting Information), *Rpa1* mRNA was increased from 12 h and sustained high level post TCR engagement. In addition to the mRNA level, we found that protein level of RPA1 was also increased in the activated T cell as compared with naïve T cells (Figure 1b and Figure S1D, Supporting Information). However, the treatment of Phorbol 12‐myristate 13‐acetate (PMA) and ionomycin that mimic T cell activation through stimulating protein kinase C and Ca^2+^ influx, elicited little effects on *Rpa1* induction in T cells (Figure 1c). Given that PMA and ionomycin treatment hardly initiates T cell proliferation, our data thus indicate the involvement of RPA1 in the modulation of T cell expansion.

### 2.2 Loss of RPA in T Cells Leads to Lymphopenia

To decipher the biological function of RPA1 in vivo, we crossed *Rpa1^fl/fl^* mice with *Cd4*‐Cre mice and generated T cell‐intrinsic *Rpa1* deficiency mice (Figure S2A, Supporting Information). These *Rpa1^fl/fl^ Cd4* ^Cre^ mice are thus referred to as conditional knockout (CKO) mice. These CKO mice are fertile and developed normally. To confirm the effectiveness of *Rpa1* deletion in CKO mice, we isolated naïve T cells from WT (*Rpa1^fl/fl^*) and CKO mice and performed western blot with anti‐RPA1 antibody. In contrast to the high level of RPA1 in WT T cells, the protein level of RPA1 was undetectable in CKO T cells (Figure 1d).

With the exception of slightly enlarged spleen in CKO mice, the lymphoid organs (lymph nodes and thymus) and nonlymphoid organ (colon) from CKO mice appear similar to those from WT mice (Figure S2B–F, Supporting Information). Through routine blood test, we found that the amount of white blood cells (WBCs), especially lymphocytes, was significantly decreased in CKO mice as compared with WT mice (Figure 1e; Figure S3, Supporting Information). We then employed flow cytometry assay to further confirm these results. As shown in Figure 1f and Figure S4A–C (Supporting Information), both the percentages and the numbers of CD4^+^ and CD8^+^ T cells were significantly reduced in spleen and lymph nodes (LNs) from CKO mice as relative to those from WT mice. Notably, compared to the 2‐fold reduction of CD4^+^ T cells, the 5‐fold reduction of CD8^+^ T cell was more pronounced in spleen from CKO mice (Figure 1f). Accordingly, the T cell survival marker CD127 (IL7R) was downregulated in both CD4^+^ and CD8^+^ CKO T cells as compared with WT T cells (Figure 1g; Figure S4D, Supporting Information).

In addition to spleen and LN, we also assess the status of T cell resident in other nonimmune organs including liver, lung and colon. As shown in Figure 1h,i, the percentages of T cells were reduced in liver and lung from CKO mice. Reciprocally, the percentages of other types of immune cells, including *γδ* T cells, B cells and myeloid cells (CD45^+^CD11c^+^ and CD45^+^CD11b^+^ cells), were increased in liver and lung from CKO mice as relative to WT mice (Figure 1j,k; Figure S5, Supporting Information). Compared with other organs, immune homeostasis in the intestine was less interrupted by RPA1 depletion (Figure 1h–k; Figure S5, Supporting Information). Above all, our data uncover that deletion of RPA1 restricts peripheral T cell population and leads to lymphopenia.

### 2.3 RPA1 Deficiency Skews TCR Repertoire of CD8^+^ T Cell

**Figure 2:**
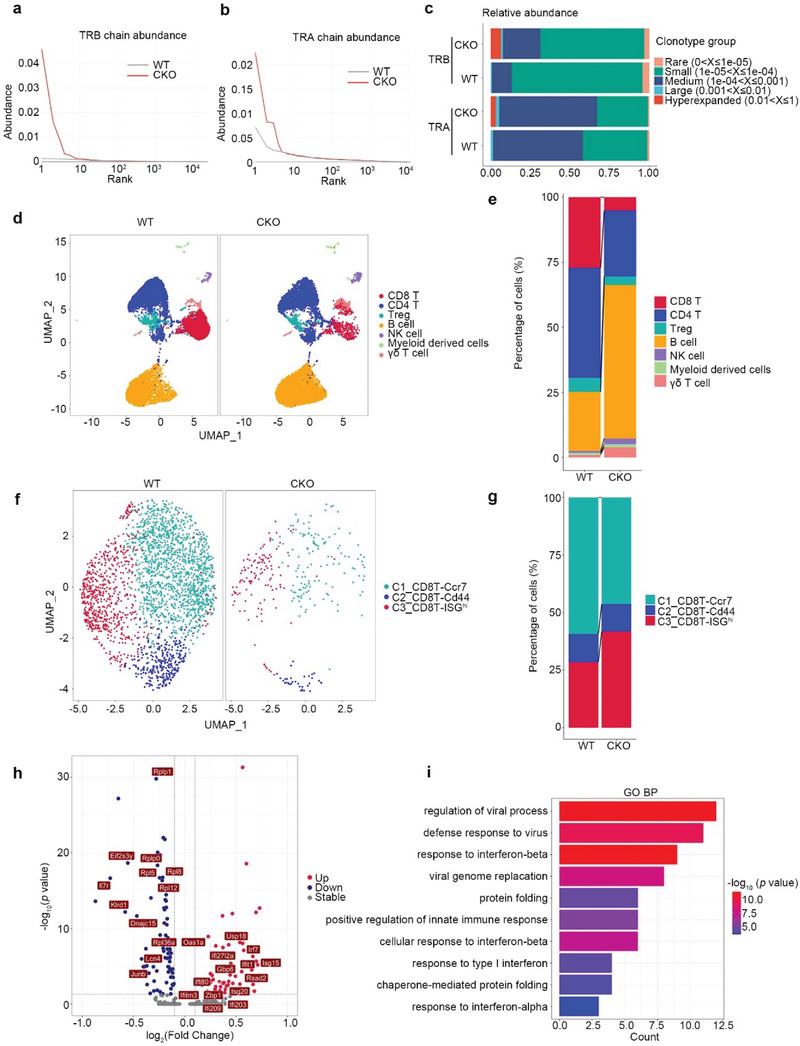
Innate immune signaling is activated in *Rpa1*‐deficient CD8^+^ T cells. a,b) The diversity analysis of CD8^+^ T cell receptors (TCR) complementarity determining region 3 (CDR3) from WT and CKO mice. The rank abundance curve of each sample in TCR *β* chain (TRB) a) and TCR *α* chain (TRA) b). The TCR clonotype is ranked by the frequency of sequencing reads. The higher the curve, the more obvious the highest frequency of TCR clonotype. c) Distribution of TCR clonotype proportions identified in CD8^+^ T cells isolated from WT and CKO mice spleen. The proportions of hyperexpanded, large, medium, small and rare clones by the percentage of next‐generation sequencing reads are shown. The increased proportion of reads taken up by the hyperexpanded and large clones indicates a lower TCR clonal diversity. d,e) CD45^+^ immune cells were isolated from lymph node (LN) in 6‐week‐old male WT and CKO mice and followed by 10 × single cell RNA‐sequencing (scRNA‐seq). UMAP plot showed the single CD45^+^ immune cells colored by 7 cell types d). Proportions of 7 cell types in WT and CKO LN were shown e). f) UMAP plot showed the single CD8^+^ T cells colored by 3 cell types. g) Proportions of 3 CD8^+^ T cell subsets in WT and CKO LN were shown. h) Volcano plots analysis of the differentially expressed genes (DEGs) between WT and CKO ISG^hi^ CD8^+^ T cells. i) DEGs between WT and CKO ISG^hi^ CD8^+^ T cells were analyzed using DAVID with Gene Ontology (GO_BP) terms.

To study whether RPA1 deficiency affects TCR repertoire, we used TCR sequencing to analyze the diversities of TCR *α*‐ and *β*‐chain in CD8^+^ T cells. We first compared the frequency distributions of TCR *β* and observed a remarkable difference between CKO and WT CD8^+^ T cells. The empirical cumulative distribution function curves showed that the frequency distribution of each private TCR *β* sequence from WT CD8^+^ T cells was shifted significantly lower than those from CKO CD8^+^ T cells (**Figure** **2**a). Similar result was also observed in the analysis of TCR *α* diversity (Figure 2b), indicating that TCR repertoire became less diversity due to RPA1 deficiency. To further analyze the basis of the diversity loss, we used the clonal space homeostasis plot to depict the TCR repertoire. As shown in Figure 2c, hyperexpanded clonotypes appeared in both TCR *α*‐ and *β*‐chain in CKO CD8^+^ T cells rather than in WT CD8^+^ T cells. Accordingly, the small and rare clonotypes was reduced in CKO CD8^+^ T cells as relative to those in WT CD8^+^ T cells (Figure 2c). Our data thus identify a reduced amount and less‐diversity TCR repertoire of CD8^+^ T cells in spleen from CKO mice.

### 2.4 Loss of RPA1 Hardly Affects T Cell Development in Thymus

Considering the essential role of thymus in T cell development, we thus assess the ratio of immune cells in thymus. As shown in Figure S6A (Supporting Information), the percentages of double negative (DN) T cells (CD4^−^CD8^−^), double positive (DP) T cells (CD4^+^CD8^+^) and single positive (SP) T cells (CD4^+^CD8^−^ or CD4^−^CD8^+^) between WT and CKO mice were identical. To further confirm this result, we applied scRNA‐seq methods to analyze CD45^+^ immune cells isolated from thymus and identified 9 immune cell clusters (Figure S6B,C, Supporting Information). Major immune cell types including DN, DN_rep_ (high‐level of genes related with DNA replication), DN_exp_ (high‐level of genes related with cell expansion), DP, CD4^int^CD8^int^, CD4^im^ (CD24^+^CD69^hi^CD4^+^CD8^−^), CD8^im^ (CD24^+^CD69^hi^CD4^−^CD8^+^), CD4SP and CD8SP were detected in both CKO and WT thymus (Figure S6B,C, Supporting Information). Interestingly, we noticed that RPA1 is highly expressed in DN_rep_ as relative to other subsets of immune cells (Figure S6D, Supporting Information). In accordance with the flow cytometry results, the percentages of various T cell subsets were identical between WT and CKO thymus (Figure S6E,F, Supporting Information). To determine whether RPA deletion affects thymocyte emigration, we measured the expression of thymocyte egress‐related genes including *Sell*, *Cd69*, *Klf2*, *Il7r*, *S1p1*, and *Ccr7* in CD4SP and CD8SP cells, represented as mature T cells. As expected, no significant difference was detected between WT and CKO mice (Figure S6G, Supporting Information). Our data thus indicate that loss of RPA1 hardly affects T cell development in thymus.

### 2.5 Loss of RPA1 Activates the Innate Immune Signaling in Peripheral T Cells

To investigate the mechanism by which RPA1 deficiency results in T cell lymphopenia, we used scRNA‐seq method to analyze the CD45^+^ immune cells isolated from CKO or WT lymph nodes (LNs). Utilizing graph‐based clustering to analyze the CD45^+^ immune cells, we identified 7 clusters of immune cells for 10 × data. We then defined the clusters based on the exclusive expression of canonical marker genes. As shown in Figure 2d and Figure S7A (Supporting Information), major immune cells including CD8^+^ T, CD4^+^ T, Treg, *γδ* T, B, NK, and myeloid cells were detected in both CKO and WT lymph nodes (LNs). Consistent with our flow cytometry data that the percentages of CD4^+^ or CD8^+^ T cells were reduced in LN from CKO mice, the ratio of *αβ* T cells (CD4^+^ or CD8^+^ T cells) was decreased in CKO LN compared with that in WT LN (Figure 2e). Considering the remarkable reduction of CD8^+^ T cells in CKO LN, we first performed unsupervised clustering and identifies three clusters for CD8^+^ T cells, each with its unique signature genes (Figure 2f and Figure S7B, Supporting Information). In contrast to the reduction of CCR7^+^ CD8^+^ T cell subset, the percentage of ISG^hi^ CD8^+^ T cells was increased in CKO LN compared with that in WT LN (Figure 2g). To clarify the potential function of RPA1 in CD8^+^ T cells, we analyzed the differentially expressed genes (DEGs) between CKO and WT ISG^hi^ CD8^+^ T cells. As shown in Figure 2h, we found that the T cell survival marker *Il7r* was downregulated in CKO ISG^hi^ CD8^+^ T cells, while the expression of ISGs including *Isg15*, *Irf7*, and *Zbp1* was increased in CKO ISG^hi^ CD8^+^ T cells as compared with control cells. We then used Gene Ontology (GO) to further analyze DEGs to assess the pathways affected by RPA1 deficiency. As shown in Figure 2i, genes related with innate immune response were enriched in CKO ISG^hi^ CD8^+^ T cells. Similar results were also detected in CKO CD4^+^ T cells (Figure S8, Supporting Information). Our data thus indicate that loss of RPA1 triggers the activation of innate immune signaling in peripheral T cells.

### 2.6 Loss of RPA1 Negatively Regulates the Survival of Recent Thymic Emigrants

**Figure 3:**
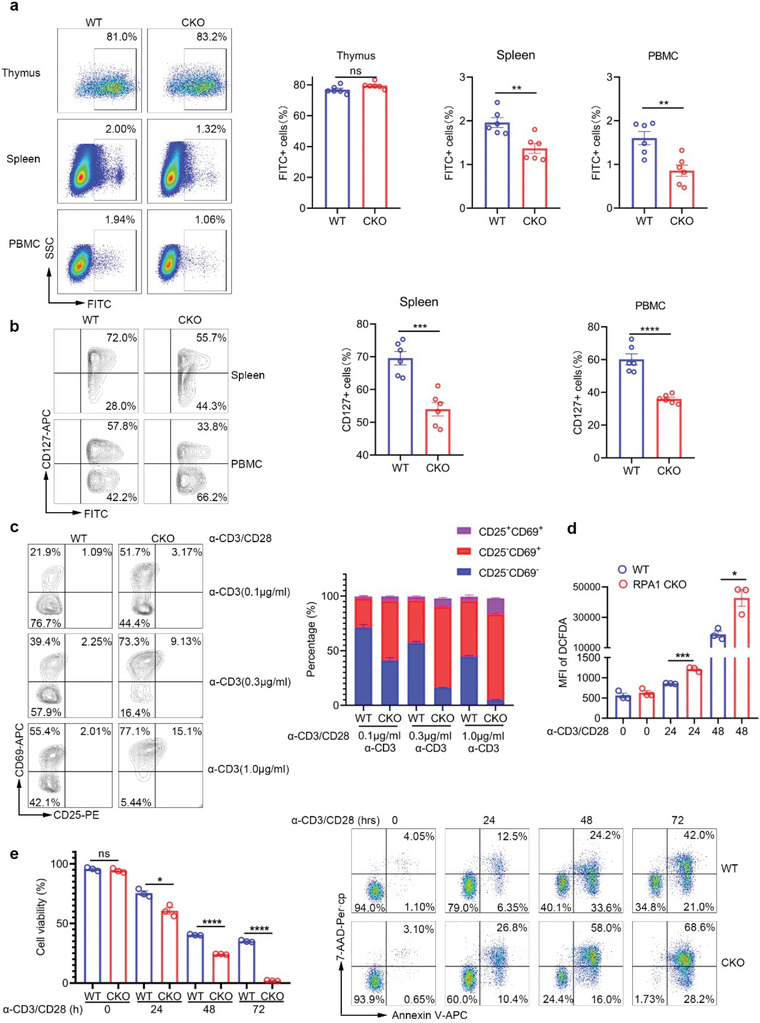
Loss of RPA1 promotes T cell death upon antigenic stimulation. a) Flow cytometric analysis of the frequencies of FITC^+^ cells in thymus, spleen and peripheral blood mononuclear cell (PBMC) from 6‐week‐old male WT and CKO mice 24 h after intrathymic injection of FITC (*n* = 6 mice, mean ± s.e.m., ns, not significant (*P* > 0.05), **P < 0.01, two‐tailed unpaired Student's *t*‐test). b) Flow cytometric analysis of the frequencies of CD127^+^ cells in FITC^+^ cells from 6‐week‐old male WT and CKO mice 24 h after intrathymic injection of FITC (*n* = 6 mice, mean ± s.e.m., ***P = 0.0003, ****P < 0.0001, two‐tailed unpaired Student's *t*‐test). c) Flow cytometric analysis of the frequencies of CD25^−^CD69^−^ cells, CD25^−^CD69^+^ cells and CD25^+^CD69^+^ cells in CD8^+^ T cells derived from 6‐week‐old male WT and CKO mice spleen. Antibodies against CD3 (0.1, 0.3, 1.0 µg mL^−1^) and CD28 (1 µg mL^−1^) were used to stimulate T cell activation (*n* = 3 biological replicates, mean ± s.e.m.). d) Flow cytometric analysis of cellular ROS level in CD8^+^ T cells derived from 6‐week‐old male WT and CKO mice spleen with anti‐CD3 (2 µg mL^−1^) and anti‐CD28 (1 µg mL^−1^) antibodies stimulation for indicated time (*n* = 3 biological replicates, mean ± s.e.m., *P = 0.0148, ***P = 0.0005, two‐tailed unpaired Student's *t*‐test). MFI, mean fluorescence intensity. e) Splenic CD8^+^ T cells derived from 6‐week‐old male WT and CKO mice were stimulated with anti‐CD3 (2 µg mL^−1^) and anti‐CD28 (1 µg mL^−1^) antibodies for indicated time. Cell viability was measured by Annexin V/7‐AAD staining (*n* = 3 biological replicates, mean ± s.e.m., ns, not significant (*P* > 0.05), *P = 0.0111, ****P < 0.0001, two‐tailed unpaired Student's *t*‐test).

Recent thymic emigrants (RTEs) are the youngest subset of naïve T cells that maintain T cell diversity in the periphery and make a particularly important contribution in the very young and in adults recovering from lymphopenia. To determine whether loss of RPA1 also affects the availability of RTEs, we used intrathymic FITC injection assay to examine the status of RTEs in mice. 24 h after intrathymic FITC injection, we used flow cytometry assay to assess the percentage of FITC^+^ cells in thymus, spleen and peripheral blood. As shown in **Figure** **3**a, the percentage of FITC^+^ cells were identical in thymus between WT and CKO mice, suggesting that identical amount of FITC were injected into each thymus. However, reduced ratio of FITC^+^ cells were observed in spleen and peripheral blood from CKO mice as compared with those from WT mice (Figure 3a). We also analyzed the expression of IL7R (CD127) on the surface of RTEs. Accordingly, lower level of CD127 was detected in CKO RTEs as compared with WT RTEs (Figure 3b), which is consistent with the result that lower expression level of CD127 in CKO naïve T cells as relative to their control cells (Figure 1g). Taken together, our data suggest that loss of RPA1 restricts the survival of RTEs and limits thymic output.

### 2.7 RPA1 Deficiency Promotes T Cell Death During Clonal Expansion

Clonal expansion of peripheral T cells, compensating for the reduced thymic output, contributes to T cell homeostasis.^[^ ^20^, ^21^ ^]^ To determine whether loss of RPA1 is involved in T cell proliferation, we used various concentration of anti‐CD3 plus anti‐CD28 antibodies to stimulate naïve T cells (CD4^+^CD25^−^CD44^lo^CD62L^hi^). As shown in Figure 3c, loss of RPA1 remarkably increased the percentages of both early (CD25^−^CD69^+^) and late (CD25^−^CD69^+^) activated T cells under different conditions. Accordingly, the production of endogenous ROS was increased in CKO CD8^+^ T cells as relative to those in WT CD8^+^ T cells (Figure 3d), indicating the stimulatory effects of RPA1 deletion on T cell activation.

We next used CFSE staining to assess the proliferative capacity of T cells. As shown in Figure S9A (Supporting Information), loss of RPA1 blocks CD8^+^ T cell expansion post stimulation with anti‐CD3/CD28 antibodies. Accordingly, the level of Ki‐67 was higher in WT CD8^+^ T cells than that in CKO CD8^+^ T cells post T cell activation (Figure S9B, Supporting Information). Rather than T cell expansion, increased cell death was observed in CKO CD8^+^ T cells following the treatment of anti‐CD3/CD28 antibodies, which was assessed by Annexin V/7‐AAD staining (Figure 3e). Our data thus indicate that loss of RPA1 not only impairs the survival of RTEs but also restricts the clonal expansion of peripheral T cells.

### 2.8 T Cell Necroptosis is Essential for RPA1 Deficiency‐Induced Lymphopenia

**Figure 4:**
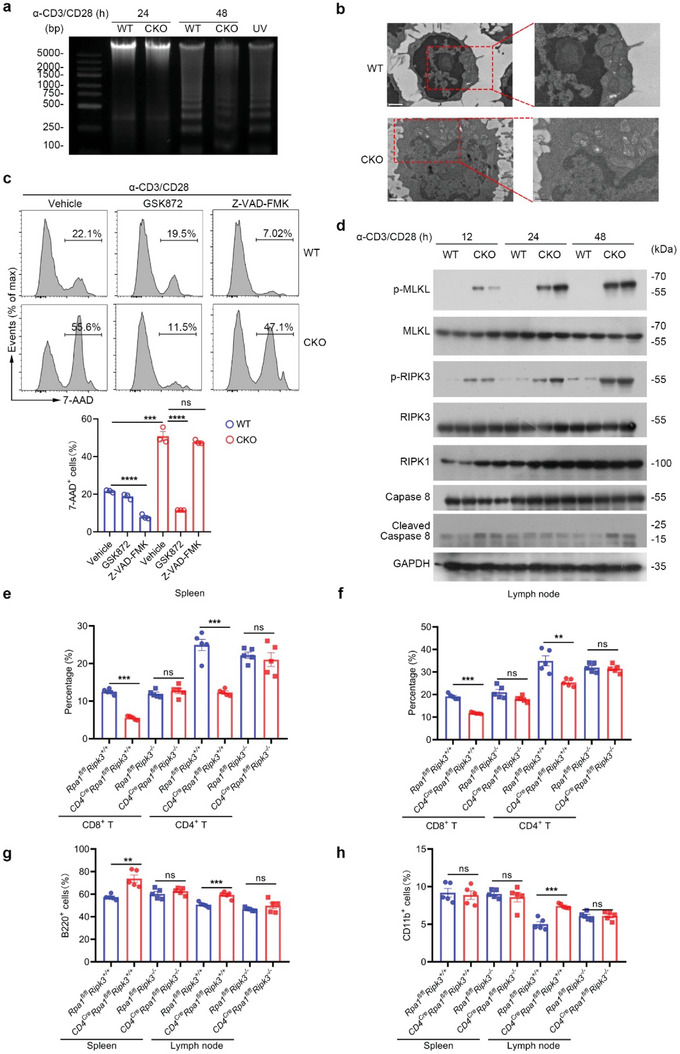
Loss of RPA1 causes peripheral T cell necroptosis post TCR engagement. a) Splenic CD8^+^ T cells derived from 6‐week‐old male WT and CKO mice stimulated with anti‐CD3 (2 µg mL^−1^) and anti‐CD28 (1 µg mL^−1^) antibodies for indicated time were harvested and prepared for the DNA fragmentation ladder assay. Naïve T cell from WT mice exposed to UV for 15 min was used as positive control. b) Representative pictures of CD8^+^ T cells derived from 6‐week‐old male WT and CKO mice spleen with anti‐CD3 (2 µg mL^−1^) and anti‐CD28 (1 µg mL^−1^) antibodies stimulation for 20 hours obtained from transmission electron microscopy (scale bars represent 1 µm). c) Splenic CD8^+^ T cells derived from 6‐week‐old male WT and CKO mice were stimulated with anti‐CD3 (2 µg mL^−1^) and anti‐CD28 (1 µg mL^−1^) antibodies for 24 h with or without cell death inhibitors. GSK872, 20 × 10^−6^ m; Z‐VAD‐FMK, 25 × 10^−6^ m. Cell viability was analyzed by 7‐AAD staining (*n* = 3 biological replicates, mean ± s.e.m., ns, not significant (*P* > 0.05), ***P = 0.0003, ****P < 0.0001, two‐tailed unpaired Student's *t*‐test). d) Immunoblot analysis of protein levels of phosphorylated MLKL, phosphorylated RIPK3, RIPK1 and cleaved caspase 8 in CD8^+^ T cells derived from 6‐week‐old WT and CKO mice spleen stimulated with anti‐CD3/CD28 antibodies for indicated times (*n* = 2 biological replicates). e,f) Flow cytometric analysis of the frequencies of indicated T cell subsets in spleen e) or lymph node (LN) f) from WT or CKO mice (*n* = 5 biological replicates, mean ± s.e.m., ns, not significant (*P* > 0.05), **P = 0.0035, ***P < 0.001, two‐tailed unpaired Student's *t*‐test). g,h) Flow cytometric analysis of the frequencies of B cells g) or CD11b^+^ myeloid cells h) in lymph node (LN) or spleen from WT or CKO mice (*n* = 5 biological replicates, mean ± s.e.m., ns, not significant (*P* > 0.05), **P = 0.0014, ***P < 0.001, two‐tailed unpaired Student's *t*‐test).

To discriminate the type of cell death induced by RPA1 deletion, we first used DNA ladder assay to study the role of RPA1 in apoptotic cell death. As shown in **Figure** **4**a, remarkable DNA ladder was observed in WT CD8^+^ T cells rather than CKO CD8^+^ T cells at 48 h post T cell activation, indicating that loss of RPA1 blocks T cell apoptosis in this process. We next employed Transmission electron microscopy (TEM) to analyze the morphological changes of WT or CKO activated T cells. As shown in Figure 4b and Figure S9C (Supporting Information), CKO activated T cells had a typical necroptotic morphology as manifested by the extensive vesiculation of cytoplasmic organelles and rupture of the plasma membrane. We thus hypothesize that loss of RPA1 can trigger T cell necroptosis. To confirm this result, we used the necroptosis inhibitor GSK872 that is selectively targeted RIPK3, to treat the activated T cells. As expected, the treatment of GSK872 rescued RPA1 deficiency‐induced T cell death following TCR engagement (Figure 4c). Conversely, the treatment of Z‐VAD‐FMK, the pan‐caspase inhibitor, elicited little effects on T cell death induced by RPA1 depletion (Figure 4c). Moreover, we employed western blot assay to assess the status of key regulators in necroptotic signaling. As shown in Figure 4d, both p‐MLKL and p‐RIPK3, which are the phosphorylated active forms of MLKL and RIPK3, were upregulated in CKO activated T cells as compared with WT control cells. However, the expression of RIPK1, caspase 8 as well as its cleaved active form were not changed in both WT and CKO activated T cells (Figure 4d). Our data thus uncover the stimulatory effects of RPA1 deficiency on T cell necroptosis.

To determine whether T cell necroptosis is the main cause for the lymphopenia induced by RPA1 deficiency, we crossed the CKO mice (*CD4^cre^Rpa1^fl/fl^*) with *Ripk3* ^−/−^ mice and generated the *CD4^cre^Rpa1^fl/fl^ Ripk3^−/−^* mice. Compared with *Rpa1^fl/fl^Ripk3^+/+^* mice, the percentages of CD4^+^ T cells and CD8^+^ T cells were decreased in both LN and spleen from *CD4^cre^Rpa1^fl/fl^Ripk3^+/+^* mice (Figure 4e,f). Conversely, homozygous deletion of RIPK3 markedly rescued the RPA1 depletion‐induced T cell suppression, as determined by flow cytometry analysis of *CD4^cre^Rpa1^fl/fl^Ripk3* ^−/−^ mice (Figure 4e,f). Accordingly, deletion of RIPK3 to some extent rebalanced the immune disorders in both LN and spleen including B cells (B220^+^) as well as myeloid cells (CD11b^+^) (Figure 4g,h). Taken together, our data demonstrate that loss of RPA1 leads to lymphopenia through triggering T cell necroptosis.

### 2.9 RPA1 Deletion Triggers DNA Damage and Activates Zbp1‐mediated Necroptotic Signaling

**Figure 5:**
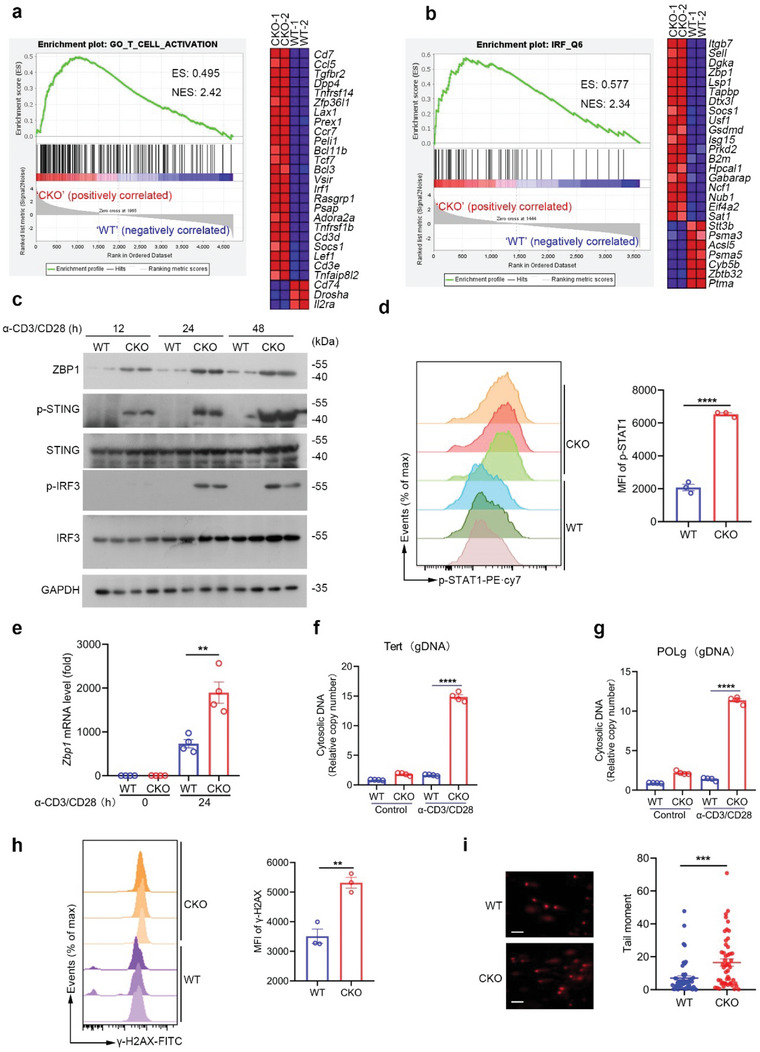
RPA1 deficiency stimulates ZBP1‐mediated necroptosis signaling. a,b) Gene set enrichment analysis (GSEA) of differentially expressed genes in CD8^+^ T cells derived from 6‐week‐old male WT and CKO mice spleen stimulated with anti‐CD3 (2 µg mL^−1^) and anti‐CD28 (1 µg mL^−1^) antibodies for 24 h. ES, enrichment score; NES, normalized enrichment score. c) Immunoblot analysis of protein levels of ZBP1, phosphorylated STING and phosphorylated IRF3 in CD8^+^ T lymphocytes derived from 6‐week‐old WT and CKO mice spleen stimulated with anti‐CD3/CD28 antibodies for indicated times (*n* = 2 biological replicates). d) Flow cytometric analysis of STAT1 phosphorylation level in CD8^+^ T cells derived from 6‐week‐old male WT and CKO mice spleen stimulated with anti‐CD3 (2 µg mL^−1^) and anti‐CD28 (1 µg mL^−1^) antibodies for 24 hours (*n* = 3 biological replicates, mean ± s.e.m., ****P < 0.0001, two‐tailed unpaired Student's *t*‐test). MFI, mean fluorescence intensity. e) RT‐qPCR analysis of mRNA level of *Zbp1* in splenic CD8^+^ T cells derived from 6‐week‐old male WT and CKO mice stimulated with anti‐CD3 (2 µg mL^−1^) and anti‐CD28 (1 µg mL^−1^) antibodies for 24 h (*n* = 4 biological replicates, mean ± s.e.m., **P = 0.0042, two‐tailed unpaired Student's *t*‐test). The primers used for RT‐qPCR have been deposited in Table S1 (Supporting Information). f,g) Splenic CD8^+^ T cells derived from 6‐week‐old male WT and CKO mice were stimulated with anti‐CD3 (2 µg mL^−1^) and anti‐CD28 (1 µg mL^−1^) antibodies for 24 h. Genomic DNA was detected by RT‐qPCR (*n* = 4 biological replicates, mean ± s.e.m., ****P < 0.0001, two‐tailed unpaired Student's *t*‐test). h) Flow cytometric analysis of H2A phosphorylation (*γ*‐H2AX) level in CD8^+^ T cells derived from 6‐week‐old male WT and CKO mice spleen stimulated with anti‐CD3 (2 µg mL^−1^) and anti‐CD28 (1 µg mL^−1^) antibodies for indicated time (*n* = 3 biological replicates, mean ± s.e.m., ns, not significant (*P* > 0.05), **P = 0.0040, two‐tailed unpaired Student's *t*‐test). MFI, mean fluorescence intensity. i) Splenic CD8^+^ T cells derived from 6‐week‐old male WT and CKO mice were stimulated with anti‐CD3 (2 µg mL^−1^) and anti‐CD28 (1 µg mL^−1^) antibodies for 48 h and subjected to alkaline comet assays. The scale bars represent 10 µm. Tail moment was analyzed by CASP Software (*n* = 50 cells, mean ± s.e.m., ***P = 0.0004, two‐tailed unpaired Student's *t*‐test).

To decipher the mechanism by which RPA1 depletion triggers T cell necroptosis, we used RNA sequencing (RNA‐seq) analysis of activated T cells in presence or absence of RPA1. Using gene set enrichment analysis (GSEA), we found that genes related with T cell activation were enriched in CKO CD8^+^ T cells (**Figure** **5**a), which is consistent with our flow cytometry data (Figure 3c). Interestingly, we noticed that interferon regulatory factor (IRF) signaling was selectively activated in CKO T cells (Figure 5b). Accordingly, the level of p‐IRF3, which is the active form of IRF3, was increased in CKO CD8^+^ T cells as compared with WT control cells (Figure 5c). Moreover, we noticed that both the IRF3 upstream (ZBP1 and STING) and downstream (p‐STAT1), were increased in CKO activated CD8^+^ T cells (Figure 5c–e).

As a Z‐DNA‐binding protein, ZBP1 has been reported to stimulate interferon‐mediated necroptotic signaling. To determine whether loss of RPA1 can result in cytosolic DNA accumulation during T cell expansion that subsequently activates ZBP1 signaling, we purified cytosolic extracts and then conducted RT‐qPCR to quantify the cytosolic DNA content in activated T cells. As shown in Figure 5f,g, a high abundance of cytosolic genomic DNA (gDNA) was observed in CKO CD8^+^ T cells as compared with those in WT control cells. Since DNA damage can trigger free‐DNA fragment production that can be released into cytosol, we thus hypothesize that loss of RPA1 can result in severe DNA damage in activated T cells. Through measurement by *γ*‐H2AX detection assay and Comet assay, we observed higher level of *γ*‐H2AX and more DNA damage was occurred in CKO CD8^+^ T cells (Figure 5h,i), which further indicates that RPA1 deletion induces severe genomic stress and DNA damage in activated T cells. Collectively, our data demonstrate that RPA1 deficiency activates ZBP1‐mediated necroptotic signaling through induction of cytosolic DNA accumulation.

### 2.10 T Cell Necroptosis by RPA1 Deletion Initiates Severe Proinflammatory Response

**Figure 6:**
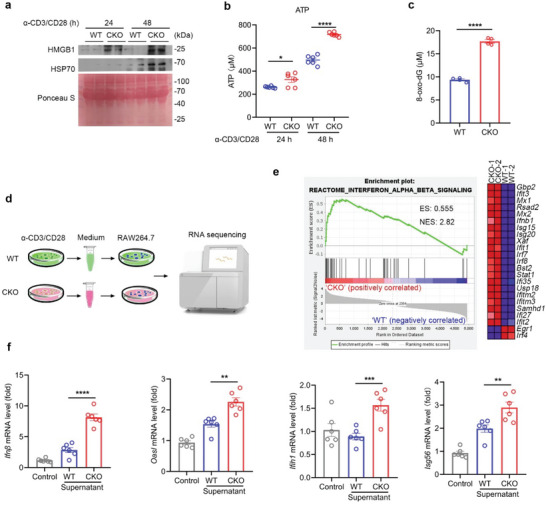
T cell necroptosis by RPA1 deletion provokes host inflammatory response. a) Splenic CD8^+^ T cells derived from 6‐week‐old male WT and CKO mice were stimulated with anti‐CD3 (2 µg mL^−1^) and anti‐CD28 (1 µg mL^−1^) antibodies for indicated time. Cell culture medium was harvested and protein levels of HMGB1 and HSP70 were measured by immunoblot analysis (*n*  = 2 biological replicates). b) Splenic CD8^+^ T cells derived from 6‐week‐old male WT and CKO mice were stimulated with anti‐CD3 (2 µg mL^−1^) and anti‐CD28 (1 µg mL^−1^) antibodies for indicated time. Cell culture medium was harvested and ATP level was measured by ATP detection kit (*n*   = 6 biological replicates, mean ± s.e.m., *P = 0.0231, ****P < 0.0001, two‐tailed unpaired Student's *t*‐test). c) Splenic CD8^+^ T cells derived from 6‐week‐old male WT and CKO mice were stimulated with anti‐CD3 (2 µg mL^−1^) and anti‐CD28 (1 µg mL^−1^) antibodies for 24 hours. Cell culture medium was harvested and 8‐oxo‐dG level was measured by enzyme linked immunosorbent assay (ELISA) (*n*  = 4 biological replicates, mean ± s.e.m., ****P < 0.0001, two‐tailed unpaired Student's *t*‐test). d,e) Splenic CD8^+^ T cells derived from 6‐week‐old male WT and CKO mice were stimulated with anti‐CD3 (2 µg mL^−1^) and anti‐CD28 (1 µg mL^−1^) antibodies for 48 hours. Cell culture medium was collected and added to RAW264.7 cell. After 24 h, RAW264.7 cells were harvested and subjected to RNA sequencing d). GSEA of differentially expressed genes in RAW264.7 cells treated with WT or CKO CD8^+^ T cell culture medium. ES, enrichment score; NES, normalized enrichment score e). f) RT‐qPCR analysis of indicated gene mRNA levels in RAW264.7 cells treated with WT or CKO CD8^+^ T cell cultures medium (*n* = 6 biological replicates, mean ± s.e.m., **P < 0.01, ***P = 0.0009, ****P < 0.0001, two‐tailed unpaired Student's *t*‐test). The primers used for RT‐qPCR have been deposited in Table S1 (Supporting Information).

In contrast to apoptosis, necroptosis acts as an immunogenic cell death that can trigger a strong inflammatory response due to the spill‐out of the cell content.^[^ ^22^ ^]^ To study whether necroptotic T cell death also leads to damage‐associated molecular patterns (DAMPs) release, we first harvested the supernatants of activated WT or CKO CD8^+^ T cells. As shown in **Figure** **6**a, greater amounts of HSP70 and HMGB1 were detected in the supernatant of activated CKO CD8^+^ T cells. Accordingly, higher level of ATP released from activated CKO CD8^+^ T cells as compared with WT CD8^+^ T cells (Figure 6b). Additionally, we found that the level of 8‐oxo‐7,8‐dihydro‐2'‐deoxyguanosine (8‐oxo‐dG), a major product of DNA oxidation, was also increased in the supernatant of activated CKO CD8^+^ T cells (Figure 6c).

To evaluate the effect of the T cell supernatant in modulation of host immune response, we thus used these supernatants to stimulate RAW264.7 cells (Figure 6d). Using RNA‐seq assay, genes related with type I interferon and cytokine signaling were enriched in RAW264.7 cells in the treatment of the activated CKO CD8^+^ T cells supernatant (Figure 6e). We also used qRT‐PCR assay to further confirm these results. As shown in Figure 6f, higher mRNA level of ISGs including *Ifnβ*, *Oasl*, *Ifih1*, *Isg56* were detected in RAW264.7 cells stimulated with the supernatant of activated CKO CD8^+^ T cells. Similar results were also detected using iBMDM cells as showed in Figure S10A (Supporting Information). Our data thus reveal that T cell necroptosis by RPA1 deletion triggers the leakage of DAMPs and provokes severe inflammatory response.

### 2.11 T Cell‐Intrinsic RPA1 Deletion Exacerbates Colonic Inflammatory Damage

**Figure 7:**
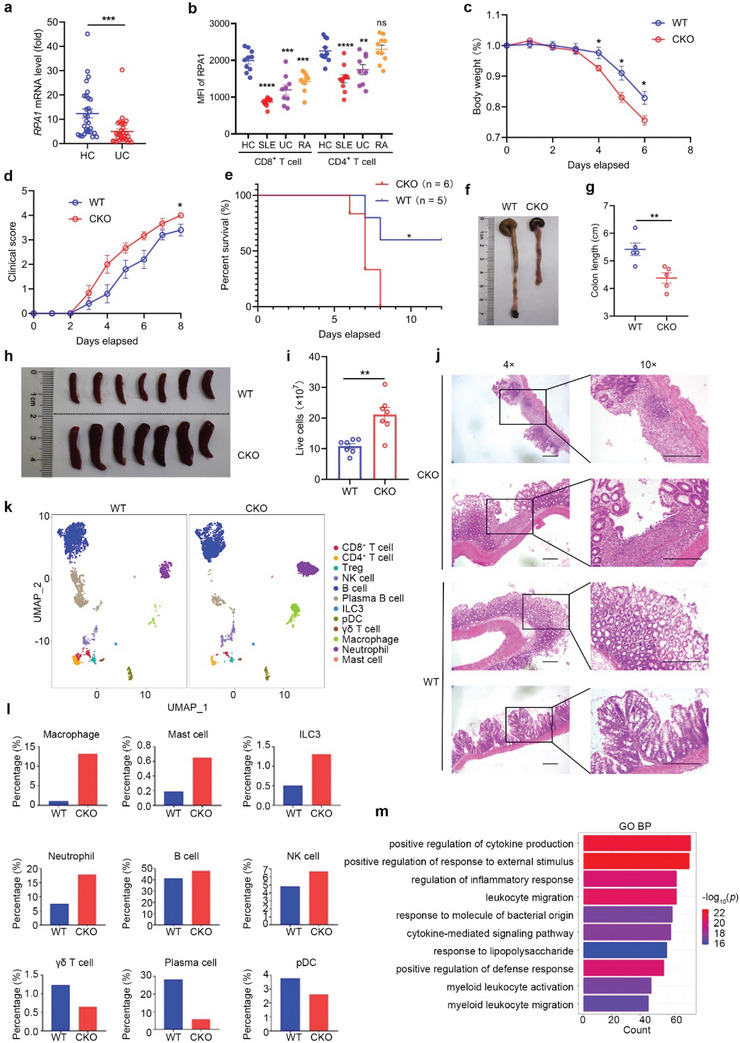
T cell‐specific Rpa1 deficient mice are more sensitive to DSS induced acute colitis. a) Quantitative real‐time PCR (RT‐qPCR) analysis of *RPA1* mRNA level in peripheral blood mononuclear cells (PBMCs) derived from ulcerative colitis (UC) patients and healthy control subjects (*n* = 30, mean ± s.e.m., ***P = 0.0010, two‐tailed unpaired Student's *t*‐test). The primers used for RT‐qPCR have been deposited in Table S1 (Supporting Information). b) The protein level of RPA1 was analyzed by flow cytometry assay. Scatter plots depicting RPA1 status in CD8^+^ and CD4^+^ T cells in peripheral blood from healthy person as well as patients with autoinflammatory diseases including SLE (Systemic lupus erythematosus), UC (Ulcerative colitis) and RA (Rheumatoid arthritis). (HC, *n* = 10; SLE, *n* = 10; UC, *n* = 9; RA, *n* = 10; mean ± s.e.m., **P < 0.005; ***P < 0.001; ****P < 0.0001; ns, no significant (*P* > 0.05), two‐tailed unpaired Student's *t*‐test). c) Body weight of 6‐week‐old male WT and CKO mice treated with 2% (weight/volume) DSS (WT, *n* = 5 mice; CKO, *n* = 6 mice; mean ± s.e.m., *P < 0.05, two‐tailed unpaired Student's *t*‐test). d) Clinical score of 6‐week‐old male WT and CKO mice treated with 2% (weight/volume) DSS (WT, *n* = 5 mice; CKO, *n* = 6 mice; mean ± s.e.m., *P = 0.0239, two‐tailed unpaired Student's *t*‐test). The colitis clinical scoring system was as follows: 0) Hard stools, fecal occult blood negative; 1‐ Feces intact, fecal occult blood positive; 2) Feces are soft or not shaped, bloody stools; 3) Loose stools, perianal blood stools; 4) Death. e) Survival analysis of 6‐week‐old male WT and CKO mice treated with 2% (weight/volume) DSS (WT, *n* = 5 mice; CKO, *n* = 6 mice; *P = 0.0323, Log‐rank (Mantel–Cox) test). f,g) Macroscopic evaluation f) and colon length g) of WT and CKO mice treated with 2% (weight/volume) DSS for 6 days (*n* = 5 mice, mean ± s.e.m., **P = 0.0077, two‐tailed unpaired Student's *t*‐test). h,i) Macroscopic evaluation h) and live cells i) of spleen from WT and CKO mice treated with 2% (weight/volume) DSS for 6 days (*n* = 7 mice, mean ± s.e.m., **P = 0.0013, two‐tailed unpaired Student's *t*‐test). j) Representative H&E staining pictures of colon tissues from WT and CKO mice treated with 2% (weight/volume) DSS for 6 days (*n* = 2 biological replicates). The scale bars represent 200 µm. k–m) CD45^+^ immune cells were isolated in colons from 6‐week‐old male WT or CKO mice treated with DSS and then analyzed by 10 × single cell RNA‐sequencing (scRNA‐seq). UMAP plot showed the single CD45^+^ immune cells colored by 12 cell types k). Proportions of 9 cell types including macrophage, mast cells, ILC3, neutrophil, B cells, NK cells, *γδ* T cells, plasma cells and pDC cells in WT or CKO LN were shown l). Differentially expressed genes between WT and CKO macrophage in colon were analyzed using DAVID with Gene Ontology (GO_BP) terms m).

Lymphopenia is tightly linked with autoimmune disorder.^[^ ^23^ ^]^ To investigate the status of RPA1 in clinical inflammatory diseases, we analyzed the transcriptional level of *RPA1* in peripheral blood mononuclear cells (PBMCs) from patients with ulcerative colitis (UC) or healthy control subjects. As shown in **Figure** **7**a, *RPA1* expression was significantly downregulated in UC patients as compared with healthy control subjects at the mRNA level. To identify the cell type in which the expression of RPA1 is downregulated, we first used the available scRNA‐seq data for PBMCs from healthy subjects and found that RPA1 is predominantly expressed in T lymphocytes (Figure S11A, Supporting Information). We next employed the flow cytometry assay with the anti‐RPA1 antibody to measure the protein level of RPA1 in both CD8^+^ T cells and CD4^+^ T cells in blood from patients with autoinflammatory diseases. As shown in Figure 7b, the protein level of RPA1 was downregulated in T cells, especially the CD8^+^ T cells, from patients with autoinflammatory diseases as relative to those in healthy persons.

To determine the involvement of RPA1 in the pathogenesis of inflammatory bowel disease, we employed dextran sulphate sodium (DSS)‐induced acute colitis mouse model. Upon exposure to 2% DSS, CKO mice exhibited increased susceptibility to experimental colitis, as determined by body weight loss, clinical score and survival time (Figure 7c–e). In accordance with the shortened colonic length (Figure 7f,g) and enlarged splenic size (Figure 7h,i) in CKO mice, histological analysis showed that severe crypt distortion, goblet cell depletion as well as massive inflammatory cell infiltration were detected in colons from CKO mice compared with wild‐type mice (Figure 7j). Furthermore, using qRT‐PCR assay, we found that higher mRNA level of ISGs including *Isg15*, *Isg56*, *Ccl5*, and *Oasl* were detected in colons from CKO mice as compared with wild‐type mice (Figure S11B, Supporting Information). In order to determine which kind of cell secretes IFN, we analyzed the CD45^+^ immune cells isolated in colon from CKO or WT mice treated with DSS by scRNA‐seq method. Utilizing graph‐based clustering to analyze the lamina propria mononuclear cell (LPMC), we identified 12 clusters of immune cells for 10 × data. We then defined the clusters based on the exclusive expression of canonical marker genes. As shown in Figure 7k, major immune cells including CD8^+^ T, CD4^+^ T, Treg, NK, B, Plasma B, ILC3, pDC, *γδ* T, Macrophage, Neutrophil, and Mast cells were detected in both CKO and WT colon. Among them, the most remarkable finding was the upregulation of macrophage in colon from CKO mice treated with DSS (Figure 7l). Further analysis of the differentially expressed genes between WT and CKO macrophage showed that genes related with positive regulation of response to external stimulus were enriched in CKO macrophage as compared with WT control cells (Figure 7m). We next analyzed the transcription of type‐I and type‐II IFN in various immune cell subsets. As shown in Figure S11C (Supporting Information), the macrophage acted as the main resource of IFN*β* production, while the IFN*γ* (type‐II IFN) was predominantly expressed in *γδ* T and NK cell, which were selectively enhanced in CKO mice.

### 2.12 Blockade of T Cell Necroptotic Signaling Reduces the Susceptibility to Experimental Colitis

**Figure 8:**
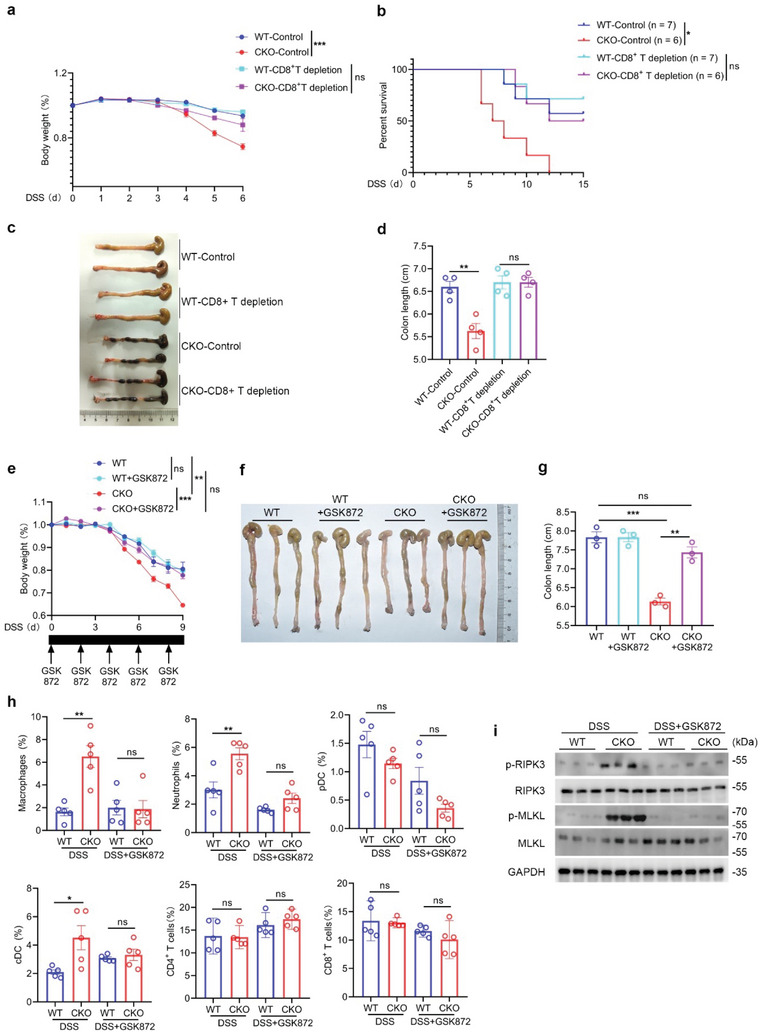
Necroptotic T cell contributes to increased susceptibility to colitis in RPA1‐deficient mice. a) Body weight of 6‐week‐old male WT and CKO mice with or without CD8^+^ T cell depletion treated with 2% (weight/volume) DSS (WT‐control, *n* = 7 mice; CKO‐control, *n* = 6 mice; WT‐CD8^+^ T depletion, *n* = 7 mice; CKO‐CD8^+^ T depletion, *n* = 6 mice; mean ± s.e.m., ***P < 0.001, two‐tailed unpaired Student's *t*‐test). b) Survival analysis of 6‐week‐old male WT and CKO mice with or without CD8^+^ T cell depletion treated with 2% (weight/volume) DSS (WT‐control, *n* = 7 mice; CKO‐control, *n* = 6 mice; WT‐CD8^+^ T depletion, *n* = 7 mice; CKO‐CD8^+^ T depletion, *n* = 6 mice; *P = 0.0122, Log‐rank (Mantel‐Cox) test). c‐d) Macroscopic evaluation c) and colon length d) of WT and CKO mice with or without CD8^+^ T cell depletion treated with 2% (weight/volume) DSS for 6 days (*n* = 4 mice; mean ± s.e.m., **P = 0.0032, ns, no significant (*P* > 0.05), two‐tailed unpaired Student's *t*‐test). e) Body weight of 6‐week‐old male WT and CKO mice treated with 2% (weight/volume) DSS with or without GSK872 (2 mg/kg) every other day (WT, *n* = 3 mice; WT+GSK872, *n* = 5 mice; CKO, *n* = 3 mice; CKO+GSK872, *n* = 5 mice; mean ± s.e.m., ns, not significant (*P* > 0.05), **P = 0.0064, ***P = 0.0007, two‐tailed unpaired Student's *t*‐test). f–g) Macroscopic evaluation f) and colon length g) of WT and CKO mice treated with 2% (weight/volume) DSS for 6 days with or without GSK872 (2 mg kg^−1^) every other day (*n* = 3, mean ± s.e.m., ns, not significant (*P* > 0.05), **P = 0.0016, ***P = 0.0006, two‐tailed unpaired Student's *t*‐test). h) Flow cytometric analysis of the frequencies of macrophage, neutrophil, pDC, cDC, CD4^+^ T cells, CD8^+^ T cells from colon in WT or CKO mice treated with DSS in presence or absence of GSK872 (*n* = 5 biological replicates, mean ± s.e.m., ns, not significant (*P* > 0.05), *P < 0.05, **P < 0.01, two‐tailed unpaired Student's *t*‐test). i) Immunoblot analysis of protein levels of phosphorylated MLKL, phosphorylated RIPK3, MLKL and RIPK3 in CD8^+^ T lymphocytes derived from 6‐week‐old WT and CKO mice treated with DSS in presence or absence of GSK872 (*n* = 3 biological replicates).

Because of the decreased amount of T cell population, the percentages of non‐T immune cells were increased in CKO mice. To exclude the contributions of reciprocally expanded non‐T immune cells in naïve CKO mice to the disease phenotypes, the anti‐CD8 neutralizing antibody was intraperitoneally administrated every fourth day after DSS treatment. As shown in **Figure** **8**a,b, depletion of CD8^+^ T cell hardly affected the inflammatory damage of WT mice as compared with control WT mice, which was determined by weight loss and survival time. Conversely, depletion of CD8^+^ T cell in CKO mice significantly improved the outcome of CKO mice with DSS‐induced acute colitis (Figure 8a,b). And the difference between WT and CKO mice became insignificant when CD8^+^ T cells were depleted, indicating that CD8^+^ T cells play an important role in the pathogenesis of DSS‐induced colonic injury of CKO mice. The ensued assessment of colon length further support the notion that CD8^+^ T cells are the cause rather than the result for the severe inflammatory damage of CKO mice (Figure 8c,d).

To determine whether T cell necroptosis is essential for the increased susceptibility of CKO mice to inflammatory damage, we treated both WT and CKO mice with DSS or DSS plus GSK872. As shown in Figure 8e–g, supplementation of GSK872 remarkably improved the outcome of CKO mice bearing acute DSS‐induced colitis, as determined by body weight loss and colon length. We then isolated immune cells in colon from WT or CKO mice treated with DSS in presence or absence of GSK872. As shown in Figure 8h, the percentages of macrophage, neutrophil as well as cDC, rather than T lymphocytes, were reduced in colons from CKO mice treated with GSK872. To determine the status of colon‐resident T cells in this process, we used western blot assay to analyze T cells with a panel of necroptosis marker antibodies. As shown in Figure 8i, the levels of phosphorylated RIPK3 (p‐RIPK3) and p‐MLKL were upregulated in T cells from CKO mice as compared with those from WT mice. Conversely, supplementation of GSK872 remarkably suppressed the phosphorylation of RIPK3 and MLKL in CKO T cells during colitis (Figure 8i). Our data thus demonstrate the importance of T cell necroptosis in the pathogenesis of colonic inflammation in CKO mice.

### 2.13 Loss of RPA1 Increases the Sensitivity of Mice to Autoimmune Hepatitis

**Figure 9:**
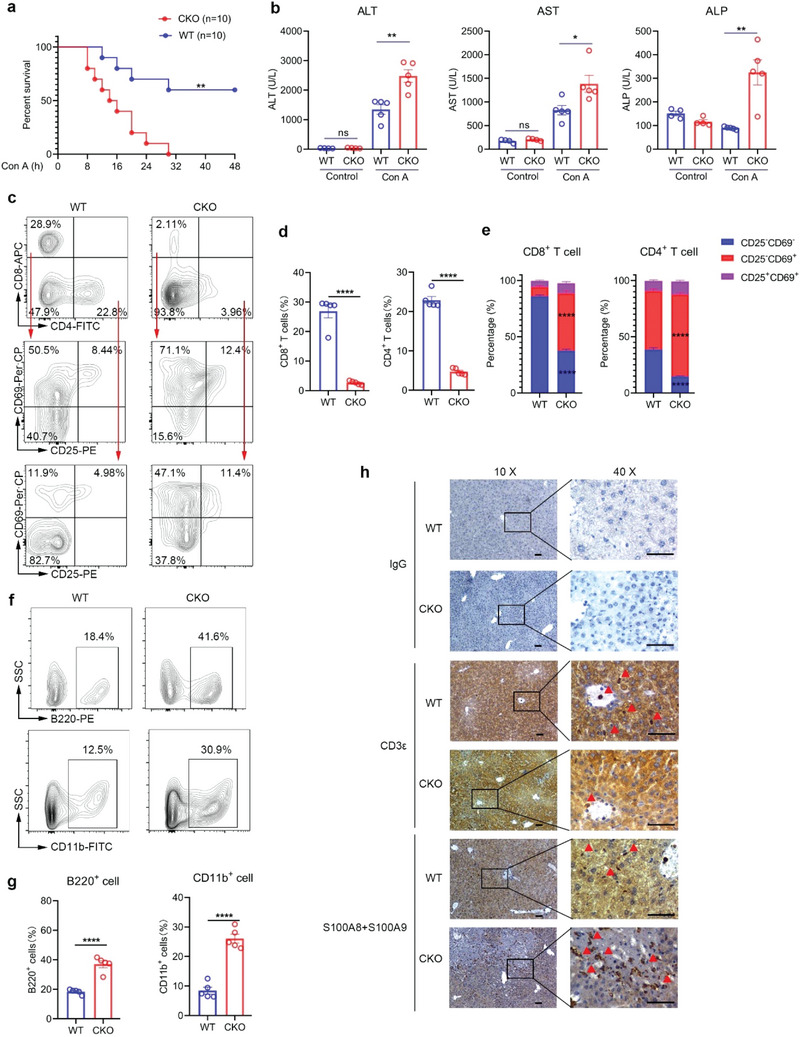
CKO mice are more sensitive to autoimmune hepatitis. a) Survival analysis of 6‐week‐old male WT and CKO mice treated with 30 mg kg^−1^ Con A (*n* = 10 mice; **P = 0.0019, Log‐rank (Mantel–Cox) test). b) 6‐week‐old male WT and CKO mice were intravenously injected with 30 mg kg^−1^ Con A and serum was collected 8 hours after injection. Serum levels of alanine aminotransferase (ALT), aspartate aminotransferase (AST) and alkaline phosphatase (ALP) were measured by Biochemical analyzer (Control, *n* = 4 mice; Con A, *n* = 5 mice; mean ± s.e.m., *P = 0.0263, **P < 0.01, two‐tailed unpaired Student's *t*‐test). c–e) Flow cytometric analysis of the frequencies of CD8^+^ T cells, CD4^+^ T cells and CD25^−^CD69^−^ cells, CD25^−^CD69^+^ cells and CD25^+^CD69^+^ cells in CD8^+^ T cells or CD4^+^ T cells derived from liver of WT and CKO mice treated with Con A for 8 h (*n* = 5, mean ± s.e.m., ****P < 0.0001, two‐tailed unpaired Student's *t*‐test). f–g) Flow cytometric analysis of the frequencies of B220^+^ cells and CD11b^+^ cells derived from liver of WT and CKO mice treated with Con A for 8 h (*n* = 5, mean ± s.e.m., ****P < 0.0001, two‐tailed unpaired Student's *t*‐test). h) Representative immunohistochemistry staining pictures of CD3*ε* and S100A8/S100A9 protein levels in liver tissues from WT and CKO mice treated with Con A for 8 h (scale bars represent 200 µm).

To explore whether T cell‐specific depletion of Rpa1 can exacerbate other inflammatory disease, we employed concanavalin A (Con A)‐induced autoimmune hepatitis mice model. As shown in **Figure** **9**a, CKO mice exhibited increased mortality compared with WT controls at 8 h after Con A administration. In spite of the identical blood biochemical index between WT and CKO mice in physiological condition, the serum levels of alanine aminotransferase (ALT), aspartate aminotransferase (AST) and alkaline phosphatase (ALP) were significantly increased in CKO mice upon exposure to Con A (Figure 9b).

We next analyzed the status of liver‐infiltrating T cells and found that the amount of T cells in liver was reduced in CKO mice as compared with their WT littermate controls in this process (Figure 9c,d). Since T cell activation is a critical process in the pathophysiology of Con A‐induced liver disease, we then used anti‐CD69 and anti‐CD25 antibodies to measure the amounts of the early or late activated T cells. As shown in Figure 9e, both CD4^+^ T cells and CD8^+^ T cells from CKO mice expressed higher level of CD25 or CD69, indicating that the threshold of T cell activation was reduced by RPA1 depletion. In addition to T cells, we also studied the percentages of other immune cells. As shown in Figure 9f,g, the ratios of CD11b^+^ myeloid cells and B220^+^ B cells were reciprocally increased in livers from CKO mice under pathological condition. To further confirm these results, we used immunohistological analysis of liver tissue with anti‐CD3e (T cell marker) or anti‐S100A8/9 (neutrophil marker). As expected, increased amounts of neutrophil and less amounts of T cells were detected in liver from CKO mice as compared with WT mice (Figure 9h). Our data thus demonstrate that T cell‐specific deletion of RPA1 increases the susceptibility of mice to autoinflammatory diseases.

## 3 Discussion

RPA1 plays a key role in DNA replication, recombination and repair in epithelial cells.^[^ ^14^ ^]^ RPA1 is also implicated in somatic hypermutation and class‐switched recombination in B cells.^[^ ^24^, ^25^ ^]^ Interestingly, recent study identified *RPA1* mutation in CD8^+^ T cells from patients with multiple sclerosis.^[^ ^26^ ^]^ However, little is known about the role of RPA1 in T cell‐mediated immune response. Our present study reports that *RPA1* transcription is downregulated in patients with ulcerative colitis. Accordingly, T cell‐intrinsic *Rpa1* depletion mice exhibit increased susceptibility to experimental colitis or hepatitis. Mechanistical study shows that loss of RPA1 triggers severe DNA damage during T cell expansion and leads to leakage of genomic DNA into cytosol, consequently activating ZBP1‐mediated necroptotic signaling. Enhanced release of DAMPs by T cell necroptosis then augments host innate immune response and exacerbates inflammatory damage (Figure S11D, Supporting Information). Our data thus uncover the essential role of RPA1 in modulation of T cell homeostasis and host inflammatory response.

Although T cell serves as a critical executor of adaptive immunity in inflammation or autoimmune disorders, T cells do not react against self‐antigens because of the presence of central and peripheral immunogenic tolerance.^[^ ^27^ ^]^ Previous studies mainly focused on the mechanism by which autoreactive T cells are generated, far less research has focused on the role of T cell death in progression of autoimmune diseases. Here, we found downregulation of RPA1 in PMBCs from colitis patients. Along with reduced TCR repertoire, loss of RPA1 triggered peripheral T cell necroptosis and therefore reduced the amounts of T cells in peripheral organs. As a form of immunogenic cell death, necroptosis can trigger host innate immune response by releasing DAMPs into surrounding microenvironment.^[^ ^28^ ^]^ Accordingly, a series of DAMPs including ATP, HMGB1 as well as 8‐oxo‐dG were leaked from RPA1‐deficient T cells upon TCR engagement, consequently initiating host inflammatory response. Conversely, blockade of T cell necroptosis by RIPK3 inhibitor largely ameliorated the inflammatory damage induced by RPA1 depletion, which further confirm the importance of T cell necroptosis in the pathogenesis of autoimmune disorders.

Robust clonal expansion is a hallmark of self‐renewal of naïve T cells, and DNA replication is the most vulnerable cellular process that can lead to T cell expansion.^[^ ^29^ ^]^ However, this process inevitably triggers errors that initiate DNA repair program, and otherwise DNA damage leads to severe replicative stress.^[^ ^11^ ^]^ As the ssDNA‐binding protein, RPA1 is essential for the initiation of DNA repair, it is therefore entirely plausible that loss of RPA1 results in severe DNA damage in activated T cells. Moreover, we found that DNA damage also led to cytosolic DNA accumulation, consequently activating ZBP1‐mediated interferon signaling. In addition to RIPK1, ZBP1 can also trigger necroptosis through activating RIPK3/MLKL signaling.^[^ ^30^ ^]^ Accordingly, we found that RIPK3/MLKL signaling rather than caspase 8/RIPK1 signaling was activated in RPA1‐deficient T cells. In light of the increased susceptibility to necroptosis in absence of RPA1, our data thus demonstrate that RPA1 is not only required for T cell expansion but also critical for maintenance of peripheral T cell pool.

Thymic T cell output is critical for the constant size of peripheral T cell pool.^[^ ^31^ ^]^ We thus focus on the role of RPA1 in the thymic T cell development. Through scRNA‐seq analysis, we find that T cell‐intrinsic deletion of RPA1 hardly affects T cell development and ensued emigration from thymus to periphery. Interestingly, we notice that RPA1 is highly expressed in CD4^−^CD8^−^ thymocytes with high level of genes related with DNA replication (DN_rep_), indicating that RPA1 is involved in the DN thymocyte proliferation. These results are consistent with data interrogated from ImmGen database (https://www.immgen.org/) ^[^ ^32^ ^]^ that high expression level of RPA1 is detected in DN4 thymocytes that undergo rapid proliferation in the subcapsular cortex. Considering the CD4‐Cre mice we used, it is conceivable that intact RPA1 is existed in DN_rep_ thymocyte from T cell‐intrinsic RPA1 deficient (CKO) mice, which partially explain our results that loss of RPA1 elicits little effects on thymic T cell development.

In contrast to the reduction of T cell numbers in other organs, the percentages of T cells in colon were almost identical between WT and CKO mice under physiological condition. To this end, we first interrogated the transcriptional data of *Zbp1* in lymph nodes (LNs), mesenteric LNs (mLNs) as well as Peyer's Patches from ImmGen database. We found the expression level of *Zbp1* is much lower in mLNs and Peyer's Patches than that in LNs. In light of the critical role of ZBP1 in the inflammatory response induced by RPA1 deficiency, it is conceivable that fewer CKO T cells in colon undergo necroptosis due to the lower level of ZBP1. Regarding the identical T cell numbers in colon between CKO and WT mice treated with DSS, we hypothesize that greater amount of T cells is recruited to the inflammatory foci because of the severe inflammatory damage in colon from CKO mice. To prove our hypothesis, WT naïve T cells were activated by anti‐CD3/CD28 antibodies for 2 days and stained by CFSE. These activated T cells were then injected into *CD4^cre^Rpa1^fl/fl^* mice and control mice treated with DSS for 6 days. Through flow cytometry analysis, we found that greater amounts of CD4^+^ and CD8^+^ T cells were recruited into colon rather than spleen in *CD4^cre^Rpa1^fl/fl^* mice. Our data thus indicate that severe inflammation in colon recruits peripheral T cells and thereby replenishes the T cell number in colon from CKO mice.

In addition to thymic output, self‐renewal of naïve T cells is also of importance for T cell replenishment.^[^ ^5^ ^]^ The cytokine IL‐7 and its receptor, IL7R, are substantial for peripheral T cell survival and proliferation.^[^ ^9^ ^]^ Previous study showed that injection of anti‐IL‐7R mAb caused a considerable decrease in the survival of T cell and blocked self‐expansion of naïve T cells.^[^ ^33^ ^]^ Consistent with those findings, naïve T cells express lower level of IL‐7R in CKO mice as compared with those did in WT mice. It has been reported that the transcription factor IRF4 plays a key role in modulation of the transcription of *CD127* gene, encoding IL7R. Moreover, innate immune signaling suppresses IRF4‐mediated gene transcription through activating the transcription factor IRF3.^[^ ^34^ ^]^ Given that the ZBP1‐TBK1‐IRF3 signaling was activated in CKO T cells during T cell expansion, it is conceivable that downregulation of IL7R is caused by activation of innate immune signaling in CKO T cells.

In summary, our data identified a previous uncharacterized role of RPA1 in modulation of T cell homeostasis. RPA1, encoded by a gene that was upregulated during T cell activation, limits DNA replication stress and promotes self‐replication of peripheral T cells, consequently contributing to the lymphocyte replenishment. Thus, our identification of the biological function of RPA1 in peripheral T cell development provides a potential therapeutic target for the treatment of inflammatory diseases.

## 4 Experimental Section

### Animals

*Rpa1^fl/fl^* mice was described previously.^[^ ^35^ ^]^ *Rpa1^fl/fl^* mice were backcrossed with C57BL/6 mice for six generations and then mated with CD4‐Cre transgenic mice to delete the floxed exons 3–4. The *Ripk3^−/^* ^−^mice were donated by Professor Wang Xiaodong (National Institute of Biological Sciences, Beijing). All animals were housed and maintained under specific pathogen‐free conditions. All sex‐ and age‐matched animal experiments were performed in accordance with protocols approved by the Ethics Committee of Peking University Health Science Center (LA2021487).

### Acute Colitis Mice Model

For induction of acute colitis, 6‐week‐old male mice were treated with 2% (weight/volume) DSS and body weights were recorded every day. The clinical score of colitis was evaluated as follows: 0‐ Hard stools, fecal occult blood negative; 1) Feces intact, fecal occult blood positive; 2) Feces are soft or not shaped, bloody stools; 3) Loose stools, perianal blood stools; 4) Death.^[^ ^36^ ^]^

### Autoimmune Hepatitis Model

For induction of autoimmune hepatitis mice model, Con A (30 mg/kg body weight in normal saline) was administered intravenously in tail vein of the 6‐week‐old male mice.

### Administration of CD8 Depletion Antibody

Anti‐CD8 antibody (Biolegend, 53–6.7) was intraperitoneally injected (10 µg/g body weight) every fourth day.

### Transmission Electron Microscopy (TEM)

WT and CKO CD8^+^ T cells were stimulated with anti‐CD3 (2 µg mL^−1^) and anti‐CD28 (1 µg mL^−1^) antibodies for 20 h. CD8^+^ T cells were fixed with 2.5% glutaraldehyde and sodium pyruvate for 24 h, post‐fixed with 2% OsO4 for 2 h, and then dehydrated. The fixed samples were subsequently cut into thin sections (60 nm). The sections were placed on formvar coated slot grids, post‐stained in uranyl acetate and Reynold's lead citrate and imaged on TEM (Hitachi, H‐600‐II).

### Immunoblot Analysis

Splenic CD8^+^ T cells were isolated from mice. Those cells were then stimulated with anti‐CD3 (2 µg mL^−1^) and anti‐CD28 (1 µg mL^−1^) antibodies or not. After T cell activation, cells were lysed by lysis buffer (10% glycerol, 0.5% NP‐40, 150 × 10^−3^ m NaCl, 0.1 × 10^−3^ m EDTA) with protease inhibitor cocktail (Roche) and subsequently subjected to SDS‐Page. Antibodies used in present study were as follows: anti‐RPA1 (abcam, ab12320), anti‐p‐MLKL (abcam, ab196436), anti‐MLKL (abcam, ab243142), anti‐caspase 8 (abcam, ab108333), anti‐cleaved caspase 8 (p18) (Immunoway, YC0108), anti‐STING (Abclonal, A3575), anti‐p‐STING (Abclonal, AP1199), anti‐RIPK1 (abcam, an202985), anti‐ZBP1 (Abclonal, A13899), anti‐p‐RIPK3 (abcam, ab195117), anti‐RIPK3 (Abclonal, A5431) and anti‐GAPDH (RayAntibody, RM2002).

### H&E Staining and IHC

For Hematoxylin‐eosin (H&E) staining, indicated tissues were fixed with formaldehyde for 48 h and then embedded with paraffin. Sections of 4 µm thick were used for H&E staining with a standard protocol. Images were acquired using an Olympus IX51 microscope. For immunohistochemistry (IHC) staining, sections of mice liver were dehydrated with graded concentrations of ethanol and endogenous peroxidase activity was blocked with 3% (v/v) hydrogen peroxide in methanol for 10 min. Antigen retrieval was carried out using 1 × 10^−3^ m EDTA buffer (PH 9.0) in a microwave oven. Sections were then incubated with antibodies against CD3*ε* (abcam, ab231775) or S100A8/S100A9 (abcam, ab288715). Sections were developed with the Envision Detection System (Dako) and counterstained with hematoxylin.

### PBMCs

PBMCs were isolated from whole blood by density gradient centrifugation using Human Lymphocyte Separation Medium (DAKEWE, 7111011). The mRNA and protein level of *RPA1* was measured in PBMCs from colitis patients or healthy persons who have signed the informed patient consent. This study was performed in accordance with the recommendations of the Declaration of Helsinki. All procedures were conducted under the approval of the Ethics Committee of Peking University Health Science Center.

### Quantitative Real‐Time PCR

Total RNA was extracted with Trizol reagent (Invitrogen) and then reverse transcribed into cDNA as previously described.^[^ ^37^ ^]^ The cDNA quantification was evaluated by ABI 7500 Detection System. The sequences of PCR primers were listed in Table S1 (Supporting Information).

### Preparation of Lymphocytes

For isolation of lamina propria mononuclear cells (LPMC), mice colon was collected and cut into 1.5 cm pieces. The pieces were incubated in HBSS with 5 × 10^−3^ m EDTA for 20 min at 37 °C. After that, epithelial cells were removed by intensive vortexing and passing through a 100 µm cell strainer. Small pieces of tissues were then placed in digestion solution containing 0.5 mg mL^−1^ Collagenase D (Roche, 11088866001) and 0.1 mg mL^−1^ DNase I (Sigma, DN25) and rotated for 40 min at 37 °C. After passed through filters, lamina propria lymphocytes were enriched through 40/80% Percoll (GE Healthcare) gradient centrifugation.^[^ ^38^, ^39^ ^]^ For isolation of liver infiltrating immune cells, murine liver was collected and cut into 1 mm^3^ pieces. After passed through filters, immune cells were enriched through density gradient centrifugation by Mouse Lymphocyte Separation Medium (DAKEWE, 7211011).

### Flow Cytometry

To detect cell surface markers, immune cells were isolated and incubated with specific antibodies for 30 min at room temperature and measured by flow cytometry. For analysis of the levels of phosphorylated STAT1 and *γ*‐H2AX, cells were fixed with 2% paraformaldehyde at 37 °C for 10 min and permeabilized with 90% ice‐cold methanol for 30 min. After washed by PBS twice, cells were incubated with antibody for 1 hour at room temperature, followed by acquisition on a flow cytometry analyzer. For intracellular ROS detection, cells were incubated with 5 × 10^−6^ m DCFDA in FBS‐free medium at 37 °C for 30 min, and then analyzed by flow cytometry. To detect the protein level of RPA1, peripheral blood mononuclear cells were isolated, fixed and permeabilized. Primary antibody against RPA1 (abcam, ab79398) and donkey anti‐rabbit IgG (Invitrogen, A21206) were used to measure RPA1 expression level. To detect recent thymic emigrants (RTEs), intrathymic FITC (10 µL of 1 mg mL^−1^ FITC solution) injection was performed by Hamilton syringe. After 24 h, immune cells of thymus, spleen and PBMC from WT and CKO mice were harvested and analyzed by flow cytometry.

Following antibodies were used: anti‐CD4 (eBioscience, GK1.5), anti‐CD8 (eBioscience, 53‐6.7), anti‐CD45 (eBioscience, 30‐F11), anti‐CD127 (BioLegend, A7R34), anti‐CD25 (BioLegend, 3C7), anti‐CD69 (I H1.2F3), anti‐B220 (BioLegend, RA3‐6B2), anti‐CD11b (BioLegend, M1/70), anti‐CD11c (BioLegend, N418), anti‐PDCA1 (eBioscience, eBio927), anti‐TCR*γ*/*δ* (BioLegend, GL3), anti‐Ly6C (BioLegend, HK1.4), anti‐Ly6G (eBioscience, RB6‐8c5), anti‐F4/80 (BioLegend, BM8), anti‐p‐STAT1 (BioLegend, A15158B), anti‐*γ*‐H2AX (BD, N1‐431), anti‐CD3 (BioLegend, 145‐2C11), and anti‐CD28 (BioLegend, 37.51).

### Routine Blood Test

The inner canthus blood was collected from 6‐week‐old male mice and subjected to the routine blood test using a HEMAVET 950FS Veterinary Multi‐species Hematology System (Drew scientific), following the manufacturer's instruction.

### ATP (Adenosine Triphosphate) Detection

Splenic CD8^+^ T cells derived from 6‐week‐old male mice were stimulated with anti‐CD3/CD28 antibodies. Cell culture medium was harvested and ATP level was measured using ATP assay kit (Nanjing Jiancheng Bioengineering Institute, A095) according to the manufacturer's protocol.

### 8‐oxo‐dG Detection

Splenic CD8^+^ T cells derived from 6‐week‐old male mice were stimulated with anti‐CD3/CD28 antibodies for 24 hours. Cell culture medium was collected and 8‐oxo‐dG level was measured by enzyme linked immunosorbent assay (ELISA) kit (MEIMIAN, MM‐45367‐M2) according to the manufacturer's protocol.

### DNA Fragmentation Ladder Assay

Splenic CD8^+^ T cells derived from 6‐week‐old male mice stimulated with anti‐CD3/CD28 antibodies were harvested and DNA fragmentation was extracted using DNA Ladder Extraction Kit with Spin Column (Beyotime, C0008), followed by 1% agarose gel electrophoresis.

### Comet Assay

Frosted microscope slides were covered with 100 µL 0.5% normal melting agarose and then maintained at 4 °C for 15 min to allow the agarose to solidify. Splenic CD8^+^ T cells derived from 6‐week‐old male mice were stimulated with anti‐CD3/CD28 antibodies for 48 h and resuspended in 0.7% lower melting agarose. The cell suspensions were pipetted onto the first agarose layer and maintained at 4 °C to allow solidification. After removal of the cover slips, the slides were incubated in cold lysis buffer (2.5 m NaCl, 100 × 10^−3^ m EDTA, 10 × 10^−3^ m Tris, 1% sodium sarcosinate, 1% Triton X‐100) at 4 °C for 1 h. The slides were then placed in a horizontal gel electrophoresis tank filled with electrophoresis solution (1 × 10^−3^ m EDTA, 300 × 10^−3^ m NaOH) for 20 min and neutralized in Tris buffer (0.4 m Tris–HCl, pH 7.5) for 20 min at 4 °C. The slides were then stained with 50 µL propidium iodide solution for 10 min. Images were acquired using an Olympus fluorescence microscope and tail moments ((Tail length × Tail% DNA)/100) were analyzed for 50 cells per slide by CASP Software.^[^ ^40^ ^]^

### Detection of Cytosolic DNA

Splenic CD8^+^ T cells derived from 6‐week‐old male WT and CKO mice were stimulated with anti‐CD3 (2 µg mL^−1^) and anti‐CD28 (1 µg mL^−1^) antibodies for 24 h. CD8^+^ T cells were divided into two equal aliquots. The whole cell extracts served as normalization controls for total gDNA. Quantitative real‐time PCR was performed on both whole cellular extracts and cytosolic fractions using gDNA primers (Tert and Plog1).^[^ ^30^ ^]^

### RNA Sequencing

To study the role of RPA1 in T cell activation, splenic CD8^+^ T cells derived from 6‐week‐old male mice were stimulated with anti‐CD3/CD28 antibodies for 24 h. To study whether loss of RPA1 induce immunogenic cell death, splenic CD8^+^ T cells derived from 6‐week‐old male mice were stimulated with anti‐CD3/CD28 antibodies for 48 h. Cell culture medium was collected and added to RAW264.7 cell. After 24 h, RAW264.7 cells were harvested and followed by RNA sequencing. Total RNA was purified using poly‐T oligo‐attached magnetic beads. RNA‐seq libraries were constructed using NEBNext Ultra RNA 24 Library Prep Kit for Illumina (NEB, USA) and sequenced on an Illumina platform with 125 bp/150 bp paired‐end reads. According to the manufacturer's instructions, clean data were obtained by removing reads containing adapter and poly‐N as well as low quality reads from raw data. Clean reads were mapped with the reference genome Hisat2 (version 2.0.5) based on the gene model annotation file. Fragments per kilobase per million mapped reads (FPKM) of each gene was calculated based on the length of the gene and reads count mapped to this gene. Gene sets from RNAseq data were analyzed for overlap with curated datasets (C5.all.V6.2, H.all.V6.2) in the MSigDB using the web interface available at http://software.broadinstitute.org/gsea/index.jsp.

### Single‐Cell RNA Sequencing

CD45^+^ immune cells were isolated from thymus, LN or LPMC by BD FACS Aria II flow cytometer. 14 000 cells were barcoded and pooled using the 10 × Genomics device. Samples were prepared following the manufacturer's protocol and sequenced on an Illumina NextSeq sequencer. To produce a raw unique molecular identifier (UMI) count matrix, the raw data were aligned and quantified using the Cell Ranger Single‐Cell Software Suite (version 5.0.0, 10 × Genomics) against the mm10 reference genome. The matrix was converted into a Seurat object by the R package Seurat (version 3.2.3). For quality control, cells with over 15% mitochondrial‐derived UMI counts were removed and 9665, 24953, or 5200 cells remained single cells were applied in downstream analyses, separately. The UMI count matrix was normalized with SCTransform function. Then, integration on two datasets was performed. In this process, potential anchors were created with FindIntegrationAnchors function of Seurat using top 3000 variable genes, and IntegrateData function was used to integrate data and create a new matrix, in which potential batch effect was regressed out. To reduce the dimensionality of the scRNA‐Seq dataset, principal component analysis (PCA) was performed. Top 40 PCs were used to perform the downstream analysis with Elbowplot function. Subsequently, t‐distributed stochastic neighbor embedding (tSNE) and uniform manifold approximation and projection (UMAP) were performed. The Seurat FindClusters function was used to divide all cells into several clusters with resolution set as 0.5, and the Seurat FindAllMarkers function was performed to identify preferentially expressed genes in clusters. The Seurat FindMarkers function was used to identify differentially expressed genes between two samples in each group. The R package dplyr (version 1.0.3) was used to count the proportion of each cell type.

### Immunosequencing

Splenic CD8^+^ T cells were isolated from mice and total RNA was extracted. TCR *α* and *β*‐chain sequences were reverse transcribed and amplified by 5′‐rapid amplification of cDNA ends (5′‐RACE) method. TCR sequencing libraries were constructed with NEBNext Ultra DNA Library Prep Kit for Illumina (NEB) and underwent quality control using Bioanalyzer High Sensitivity DNA chip (Agilent). TCR *α* and *β*‐chain libraries were sequenced on Illumina Miseq platform.

### Statistical Analysis

Statistical analysis was performed using Prism GraphPad software v8.0. Differences between two groups were calculated using a two‐tailed Student's *t* test. Mice survival status was analyzed by Log‐rank (Mantel‐Cox) test. *P < *0.05 was considered significant.

## Conflict of Interest

The authors declare no conflict of interest.

## Author Contributions

J.S. and D.L. conceived and designed the experiments; J.S. and Xin Z. performed most of the experiments and analyzed the data; Yue Y., M.G., L.W., C.R., and Yuxin Y. assisted in some experiments; Xuyang Z. and Xuehui Z. performed mass spectrometry analysis; D.L. and X.D. and J.S. wrote the paper. J.S. and D.L. provided funding. Xuehui Z., Yuxin Y., X.D., and D.L. supervised biological research and the study design.

## Supporting information

Supporting Information

## Data Availability

The data that support the findings of this study are available from the corresponding author upon reasonable request.
