# Supplementary material for: Loss of RPA1 Impairs Peripheral T Cell Homeostasis and Exacerbates Inflammatory Damage through Triggering T Cell Necroptosis
Source: Adv Sci (Weinh). 2023 Jan 31;10(11):2206344. doi: 10.1002/advs.202206344 (PMC10104672; doi:10.1002/advs.202206344)
Supplement: Supplementary file 1 — Supporting Information [file ADVS-10-2206344-s001.pdf]

ADVANCED SCIENCE
Open Access

Supporting Information

for *Adv. Sci.*, DOI 10.1002/advs.202206344

Loss of RPA1 Impairs Peripheral T Cell Homeostasis and Exacerbates Inflammatory Damage through Triggering T Cell Necroptosis

Jia Song, Xin Zhang, Yue Yin, Mengfan Guo, Xuyang Zhao, Likun Wang, Caixia Ren, Yuxin Yin, Xuehui Zhang*, Xuliang Deng* and Dan Lu**

Supporting Information

Loss of RPA1 impairs peripheral T cell homeostasis and exacerbates inflammatory damage through triggering T cell necroptosis

Jia Song, Xin Zhang, Yue Yin, Mengfan Guo, Xuyang Zhao, Likun Wang, Caixia Ren, Yuxin Yin, Xuehui Zhang, Xuliang Deng and Dan Lu

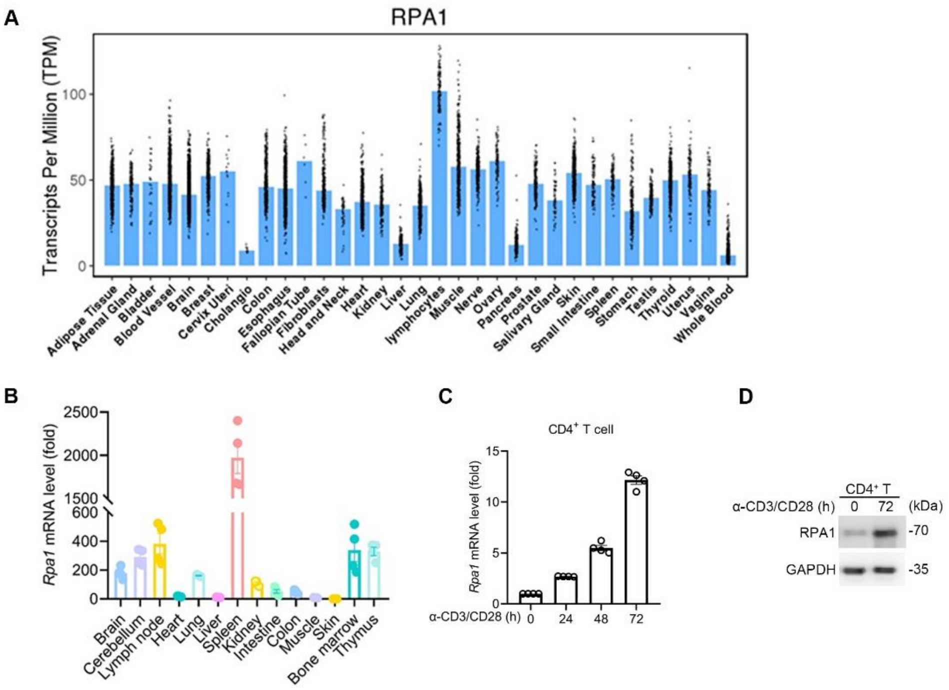

Figure S1. RPA1 is upregulated during T cell expansion.

A) Tissues distribution of RPA1 analyzed with GE-mini database (http://gemini.cancer-pku.cn/).

B) Quantitative real-time PCR (RT-qPCR) analysis of the mRNA level of *Rpa1* in indicated mouse tissues (n = 4 mice, mean ± s.e.m.). The primers used for RT-qPCR have been deposited in Table S1.

C) RT-qPCR analysis of *Rpa1* mRNA level in naïve CD4$^+$ T cells derived from 6-week-old wild-type (WT) mice spleen. Antibodies against CD3 (2 µg/mL) and CD28 (1 µg/mL) were used to activate naïve T cells (n = 4 biological replicates, mean ± s.e.m.). The primers used for RT-qPCR have been deposited in Table S1.

D) Immunoblot analysis of protein level of RPA1 in naïve CD4$^+$ T cells derived from 6-week-old WT mice spleen with or without anti-CD3/CD28 antibody treatment.

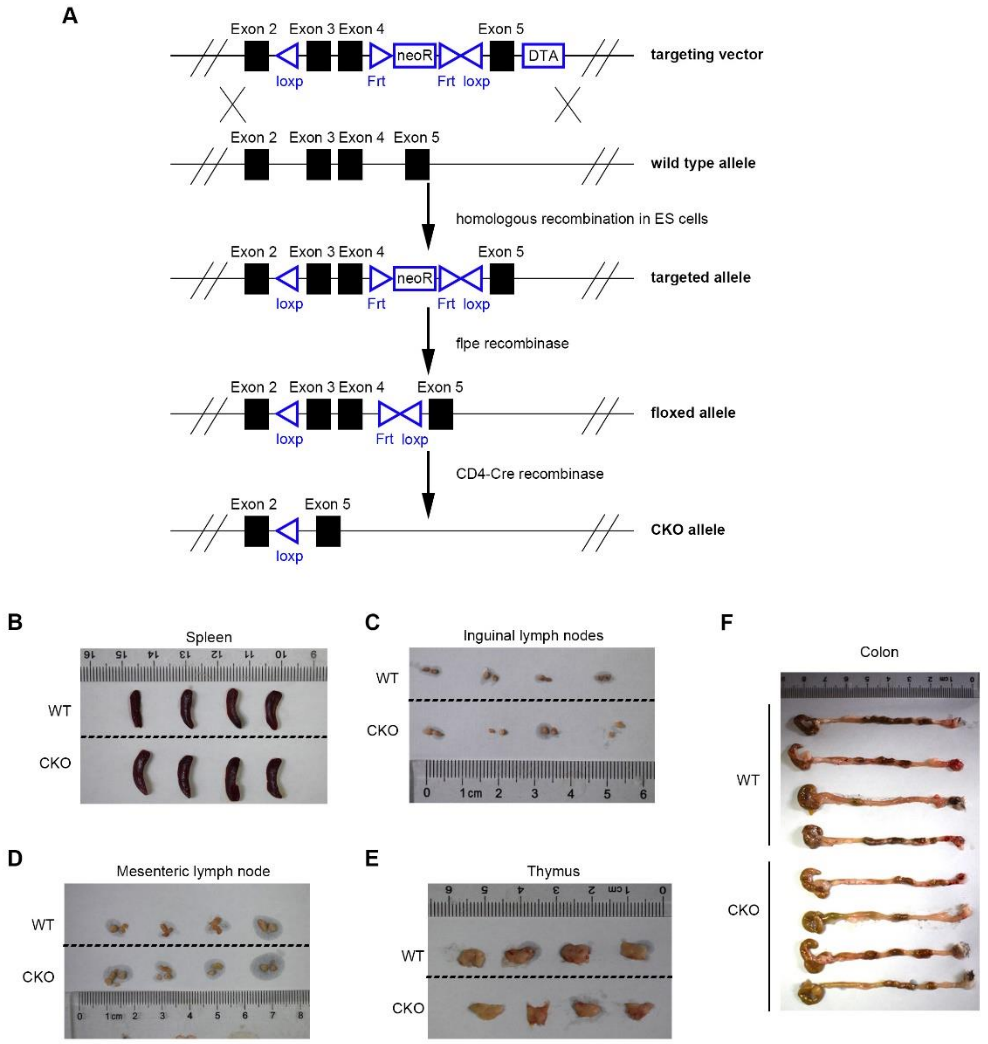

Figure S2. Generation of *Rpa1* conditional knockout mice.

A) Schematic diagram of strategy for generation of T cell-specific *Rpa1* deficient mice.

B-F) Gross tissue evaluation of spleen (B), inguinal lymph nodes (C), mesenteric lymph nodes (D), thymus (E) and colon (F) from 6-week-old male WT and CKO mice (n = 4 mice).

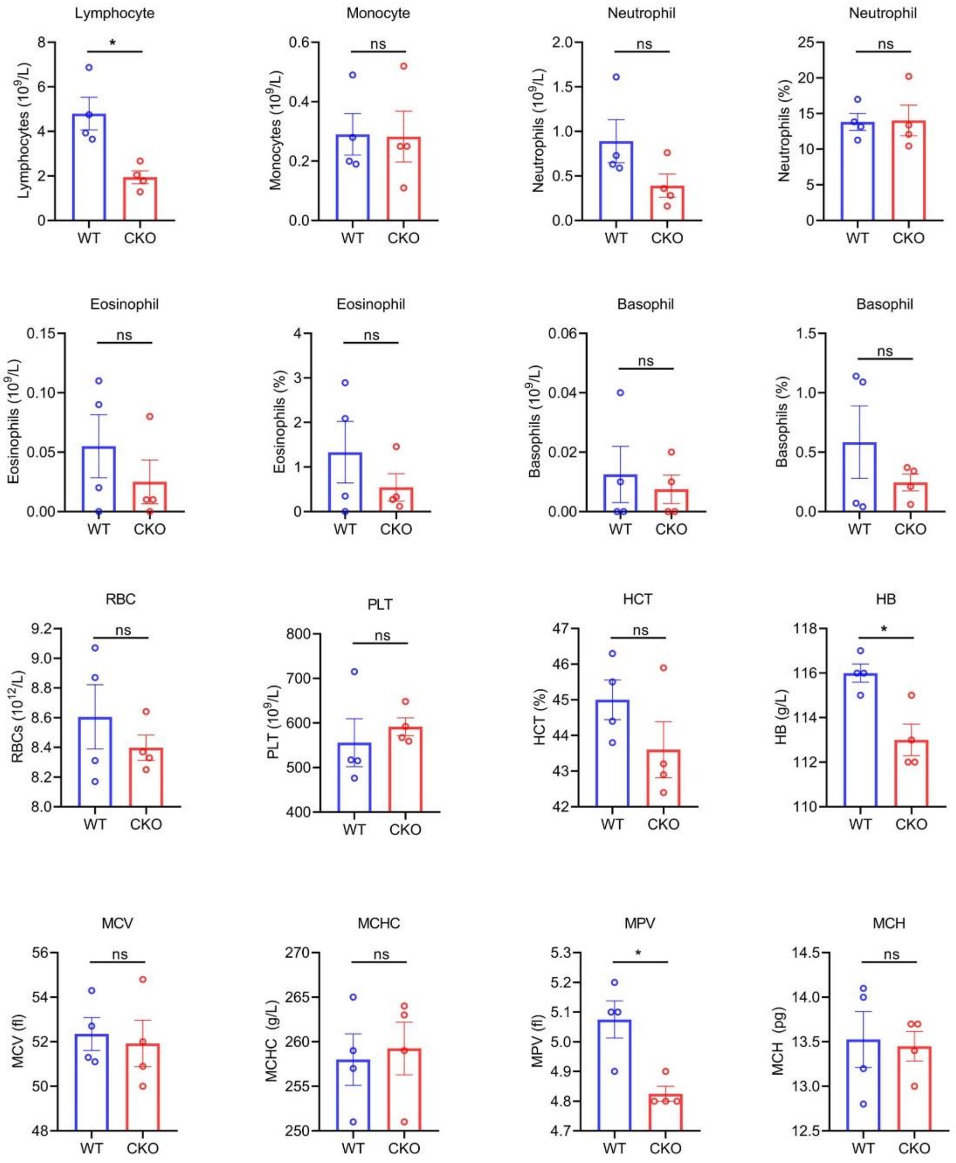

Figure S3. Routine blood test of WT and RPA1 CKO mice.

Quantity of lymphocytes, monocytes, neutrophils, eosinophils, basophils, red blood cells (RBCs), platelet (PLT), hematocrit (HCT), hemoglobin (HB), mean corpusular volume (MCV), mean corpusular hemoglobin concerntration (MCHC), mean platelet volume (MPV) and mean corpusular hemoglobin (MCH) in 6-week-old male WT and CKO mice were analyzed by routine blood test using a HEMAVET 950FS Veterinary Multi-species Hematology System (n = 4 biological replicates, mean ± s.e.m., ns, not significant ($P > 0.05$), *$P < 0.05$, two-tailed unpaired Student's t-test).

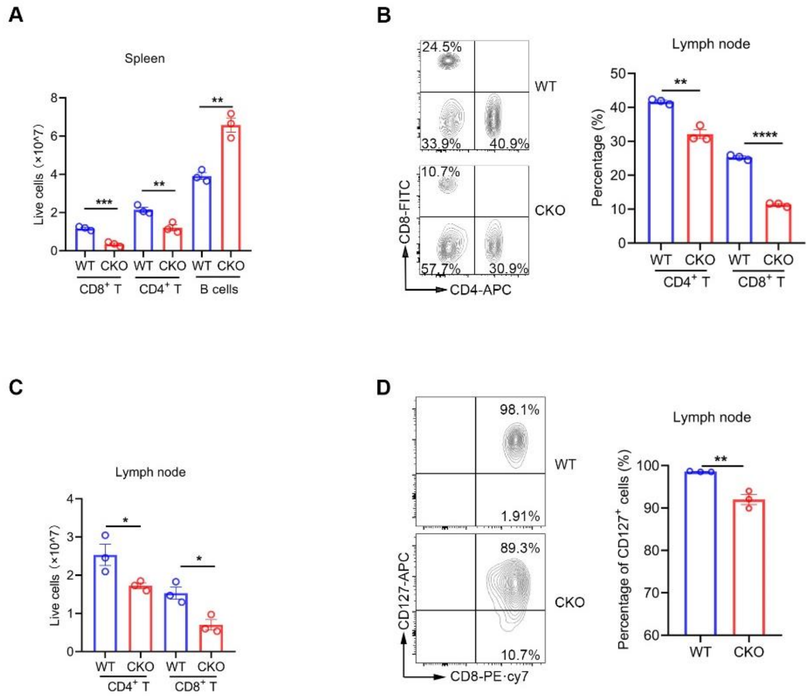

Figure S4. The status of immune cells from spleen or lymph node in WT and RPA1 CKO mice.

A) Flow cytometric analysis of the numbers of $CD4^+$ T cells, $CD8^+$ T cells and B cells from spleen in 6-week-old male WT or CKO mice (n = 3 biological replicates, mean ± s.e.m., $^{**}P < 0.01$, $^{***}P = 0.0009$, two-tailed unpaired Student's t-test).

B) Flow cytometric analysis of the frequencies of $CD4^+$ T cells and $CD8^+$ T cells in lymph node (LN) from 6-week-old male WT and CKO mice (n = 3 mice, mean ± s.e.m., $^{**}P = 0.0022$, $^{****}P < 0.0001$, two-tailed unpaired Student's t-test).

C) Flow cytometric analysis of the numbers of $CD4^+$ T cells and $CD8^+$ T cells from lymph node in 6-week-old male WT or CKO mice (n = 3 biological replicates, mean ± s.e.m., $^{*}P < 0.05$, two-tailed unpaired Student's t-test).

D) Flow cytometric analysis of $CD127^+$ cells frequency in $CD8^+$ T cells derived from 6-week-old male WT and CKO mice lymph node (n = 3 mice, mean ± s.e.m., $^{**}P = 0.0052$, two-tailed unpaired Student's t-test).

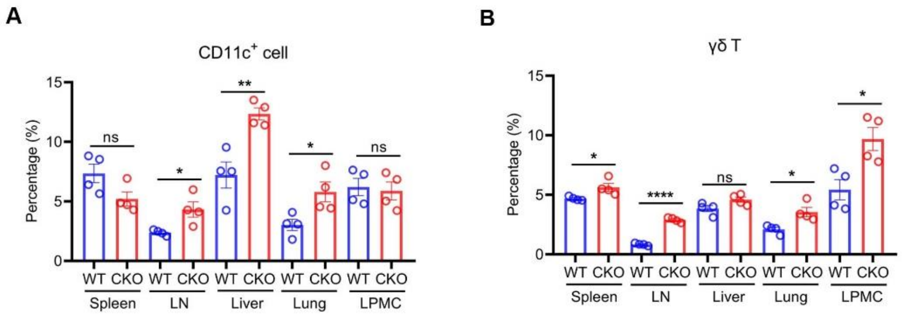

Figure S5. The status of innate immune cells in WT and RPA1 CKO mice.

A-B) Flow cytometric analysis of the frequencies of CD11c$^+$ cells (n = 4 mice, mean ± s.e.m., ns, not significant (*P* > 0.05), **P* < 0.05, ***P* = 0.0054, two-tailed unpaired Student's t-test) (A) and γδ T cells (n = 4 mice, mean ± s.e.m., ns, not significant (*P* > 0.05), **P* < 0.05, *****P* < 0.0001, two-tailed unpaired Student's t-test) (B) in spleen, lymph node (LN), liver, lung and lamina propria mononuclear cell (LPMC) from 6-week-old male WT and CKO mice.

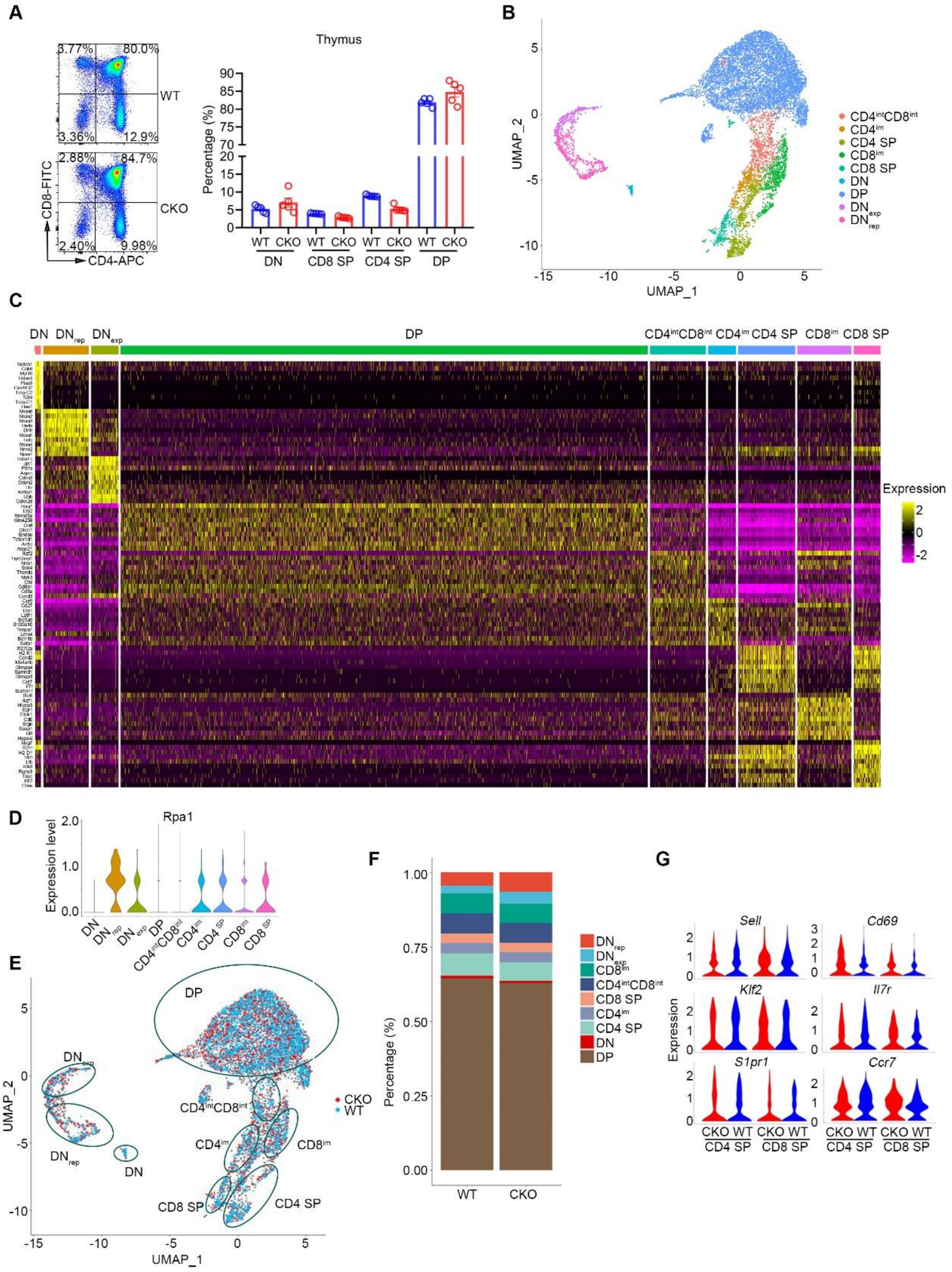

Figure S6. Single cell RNA sequencing analysis of immune cells in thymus from WT and CKO mice.

A) Flow cytometric analysis of the frequencies of T cell subsets in thymus from

6-week-old male WT and CKO mice (n = 5 mice, mean ± s.e.m.).

B-G) $CD45^+$ immune cells were isolated from thymus in 6-week-old male WT and CKO mice and followed by 10× single cell RNA-sequencing (scRNA-seq). UMAP plot showed the single $CD45^+$ immune cells colored by 9 cell types (B). Heatmap of the classic marker gene expression in 9 immune cell subsets was shown (C). Violin plots of *Rpa1* mRNA level in 9 cell types (D). Proportions of 9 cell types in WT and CKO mice thymus were shown. Red, CKO; blue, WT (E-F). Violin plots of indicated gene mRNA levels in CD4 SP and CD8 SP cells derived from WT and CKO mice thymus (G).

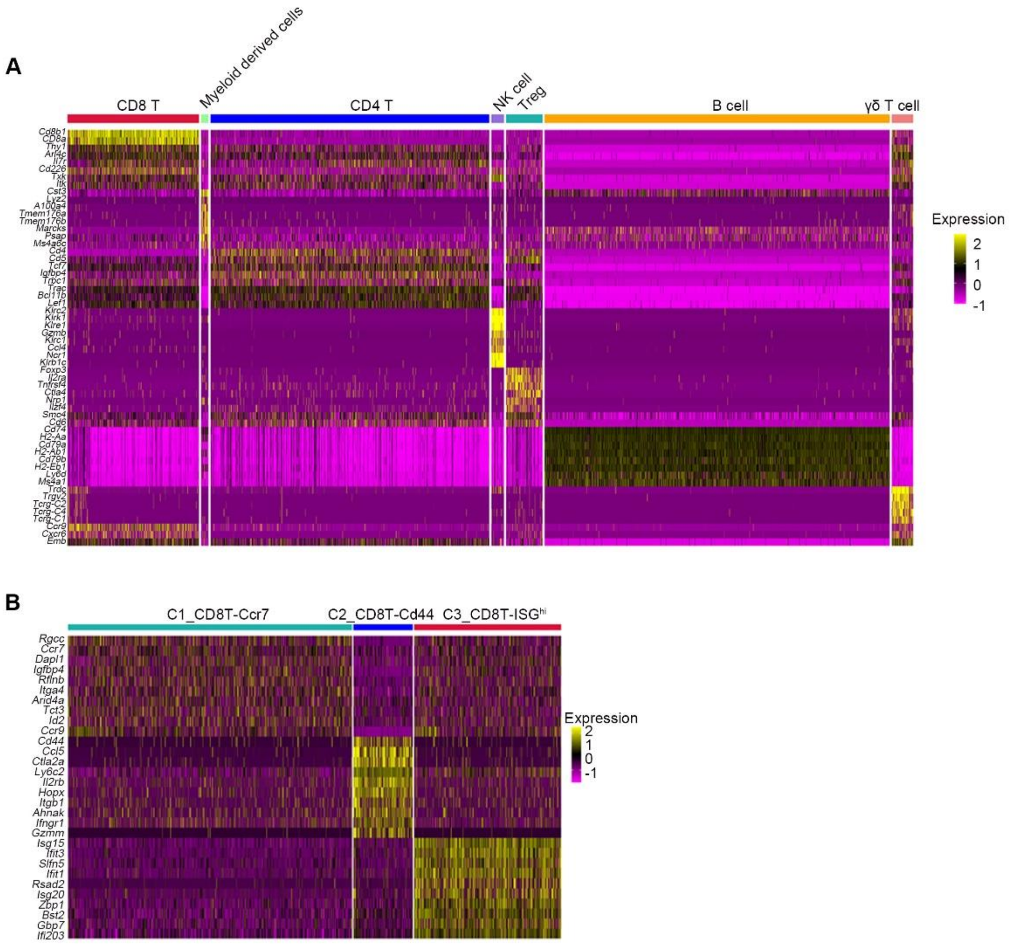

Figure S7. Heatmap of the classic marker gene expression for scRNA-seq data.

A) $CD45^+$ immune cells were isolated from lymph node (LN) in 6-week-old male WT and CKO mice and followed by 10× single cell RNA-sequencing (scRNA-seq). Heatmap of the classic marker gene expression in 7 immune cell subsets was shown.

B) Heatmap of the classic marker gene expression in 3 $CD8^+$ T cell subsets was shown.

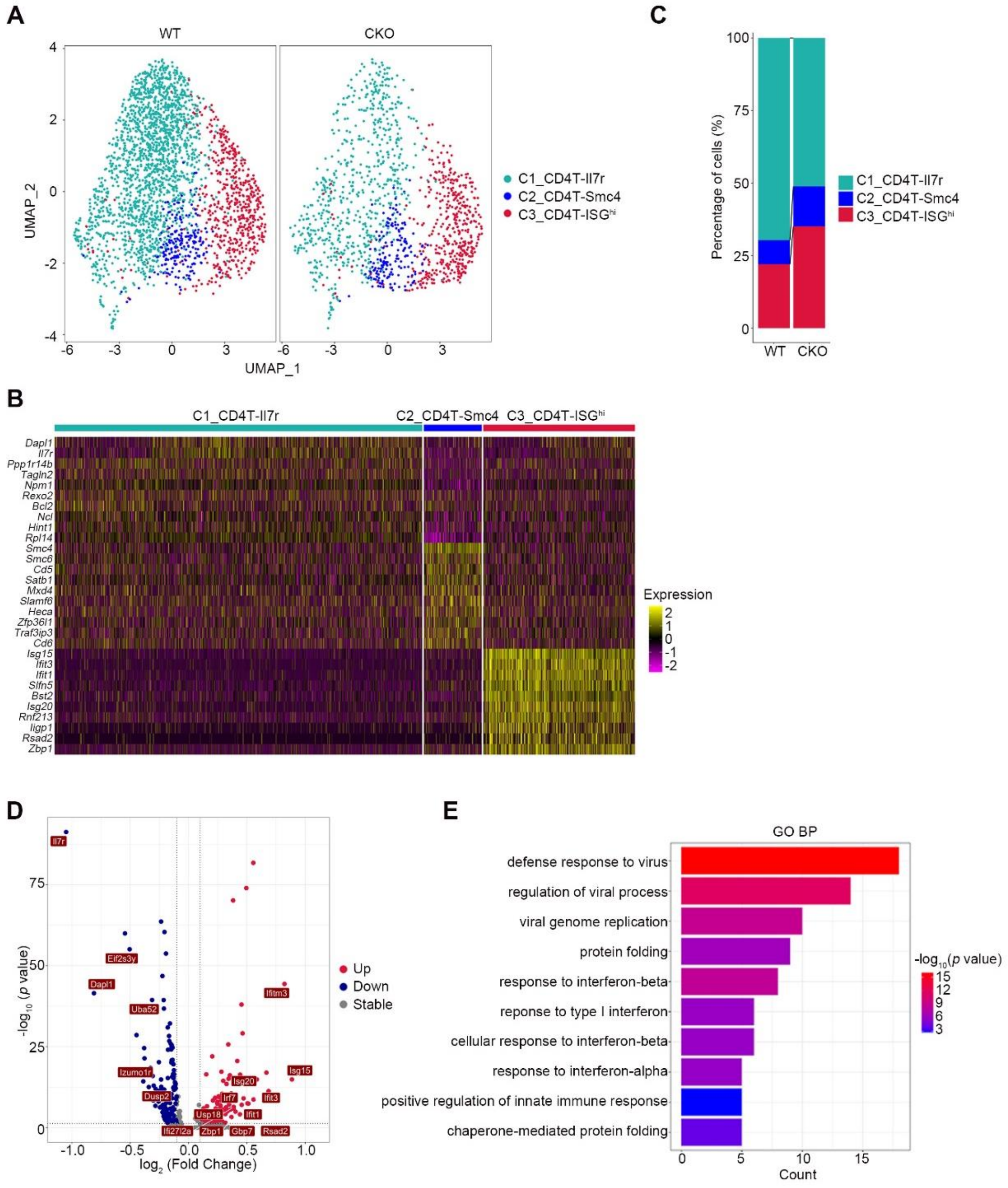

Figure S8. Innate immune signaling is activated in *Rpa1*-deficient CD4$^+$ T cells.

A) UMAP plot showed the single CD4$^+$ T cells colored by 3 cell types.

B) Heatmap of the classic marker gene expression in 3 CD4$^+$ T cell subsets was shown.

C) Proportions of 3 CD4$^+$ T cell subsets in WT and CKO LN were shown.

D) Volcano plots analysis of the differentially expressed genes (DEGs) between WT

and CKO ISG^{hi} CD4^{+} T cells.

E) DEGs between WT and CKO ISG^{hi} CD4^{+} T cells were analyzed using DAVID with Gene Ontology (GO_BP) terms.

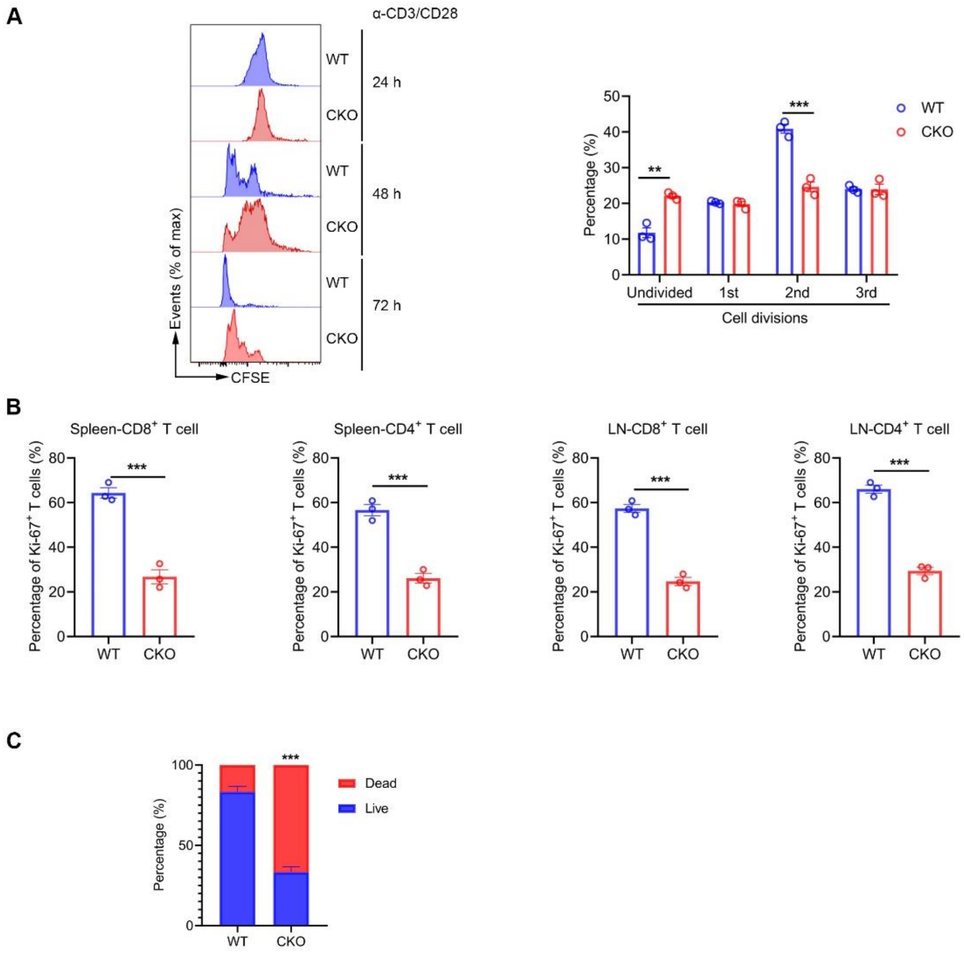

Figure S9. Loss of RPA1 blocks T cell proliferation.

A) CD8$^+$ T cells derived from 6-week-old male WT and CKO mice spleen were stimulated with anti-CD3 (2 μg/mL) and anti-CD28 (1 μg/mL) antibodies for indicated time. Cell proliferation was measured by CFSE staining and statistics of cell divisions at 48 hours was shown (n = 3 biological replicates, mean ± s.e.m., $^{**}P = 0.0022$, $^{***}P = 0.0008$, two-tailed unpaired Student's t-test).

B) Flow cytometric analysis of Ki-67$^+$ cells frequency in T cell subsets derived from 6-week-old male WT and CKO mice spleen and lymph node stimulated with anti-CD3 (2 μg/mL) and anti-CD28 (1 μg/mL) antibodies for 24 hours (n = 3 biological replicates, mean ± s.e.m., $^{***}P < 0.001$, two-tailed unpaired Student's

t-test). MFI, mean fluorescence intensity.

C) Statistical analysis of the data obtained by transmission electron microscopy for three independent exprerіments.

A

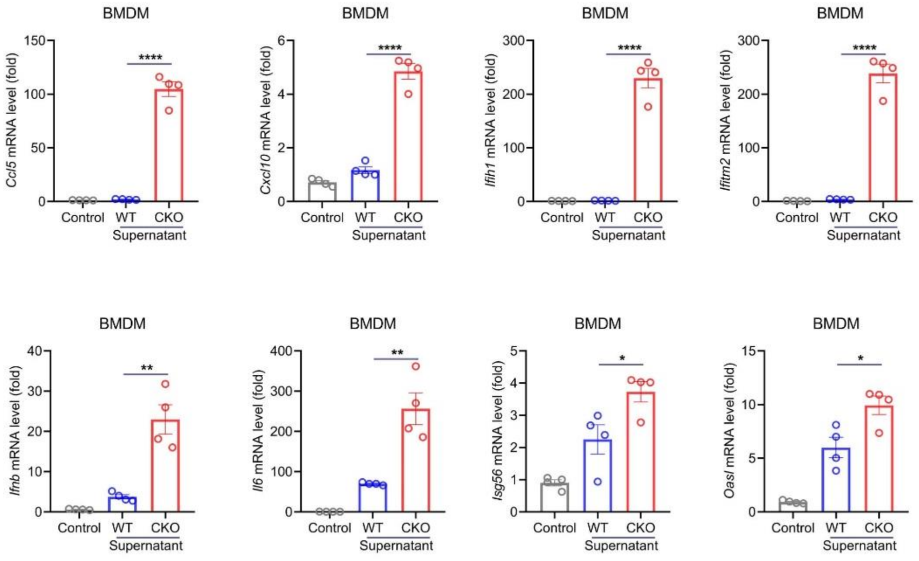

Figure S10. Loss of RPA1 promotes host inflammatory response.

A) RT-qPCR analysis of indicated gene mRNA levels in BMDM cells treated with WT or CKO CD8$^+$ T cell cultures medium (n = 4 biological replicates, mean ± s.e.m., **P* < 0.05, ***P* < 0.01, *****P* < 0.0001, two-tailed unpaired Student's t-test). The primers used for RT-qPCR have been deposited in **Table S1**.

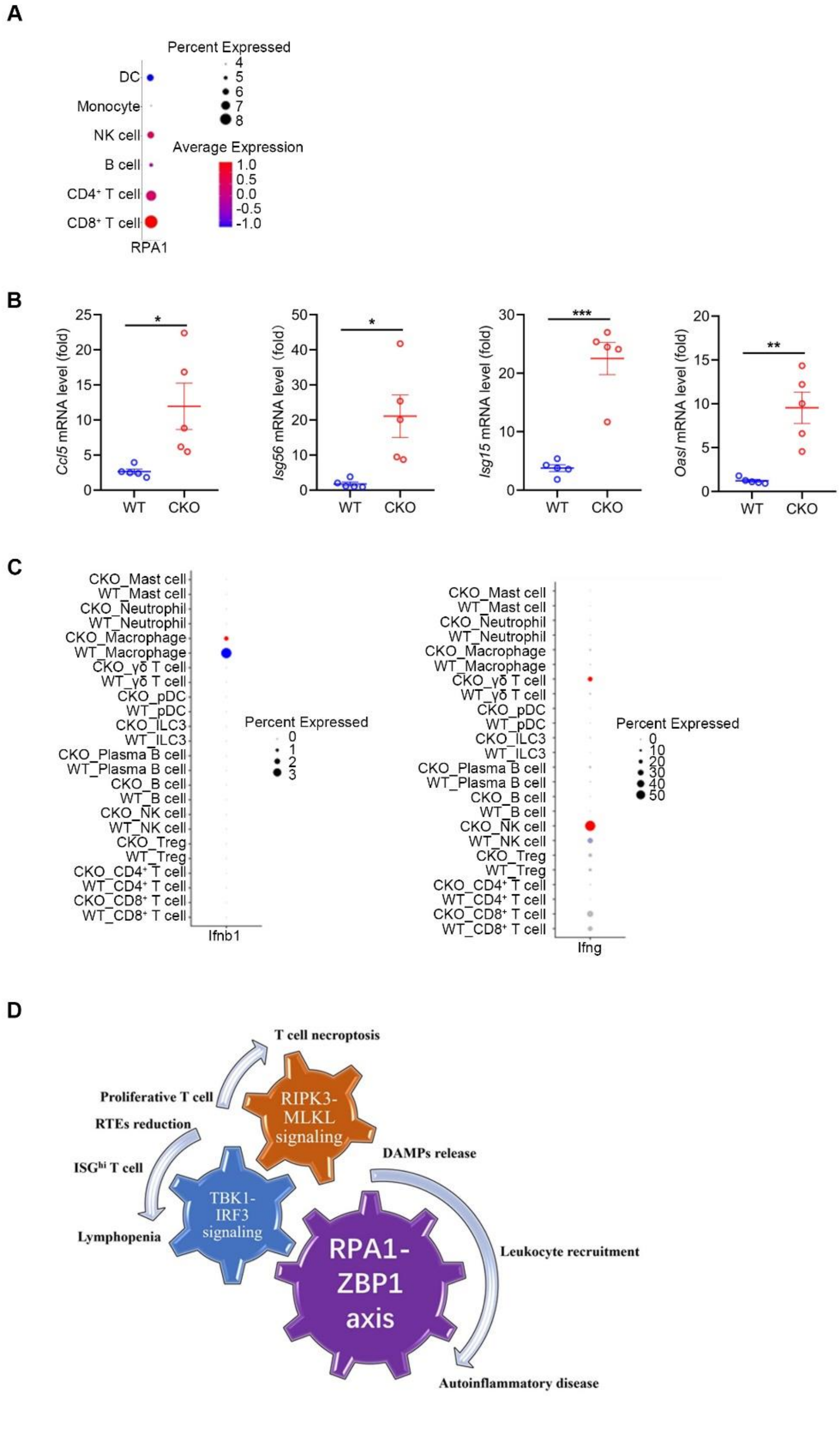

Figure S11. *Rpa1*-deficient mice exhibit increased susceptibility to experimental colitis.

A) Dot plot showed the expression of RPA1 in various immune cells. Size indicates the percent expressed cells and color indicates the average expression of RPA1 in

various cell types.

B) RT-qPCR analysis of indicated mRNA levels in colon from WT or CKO mice treated with 2% (weight/volume) DSS for 6 days (n = 5 biological replicates, mean ± s.e.m., *$P < 0.05$, **$P = 0.0017$, ***$P = 0.0002$, two-tailed unpaired Student's t-test). The primers used for RT-qPCR have been deposited in Table S1.

C) Dot plot showed the expression of IFNβ (Left) or IFNγ (Right) in various WT or CKO immune cells. Size indicates the percent expressed cells and color indicates the average expression of indicated genes in various cell types.

D) Graphic model of the role of RPA1 in modulation of T cell homeostasis and host autoinflammatory response.

Table S1. The primers used for quantitative real-time PCR were as follows:

Primer name	Sequence (5'->3') -Forward	Sequence (5'->3') -Reverse
Rpa1	ACATCCGTCCCATTTCTACAGG	CTCCCTCGACCAGGGTGTT
Zbp1	AAGAGTCCCCTGCGATTATTTG	TCTGGATGGCGTTTGAATTGG
Ifnβ	CAGCTCCAAGAAAGGACGAAC	GGCAGTGTAACTCTTCTGCAT
Oasl	CAGGAGCTGTACGGCTTCC	CCTACCTTGAGTACCTTGAGCAC
Ifih1	AGATCAACACCTGTGGTAACACC	CTCTAGGGCCTCCACGAACA
Isg56	CTGAGATGTCACTTCACATGGAA	GTGCATCCCCAATGGGTTCT
Isg15	GGTGTCCGTGACTAACTCCAT	TGGAAAGGGTAAGACCGTCCT
Ifitm2	TGGGCTTCGTTGCCTATGC	AGAATGGGGTGTTCTTTGTGC
Il6	TAGTCCTTCCTACCCCAATTTCC	TTGGTCCTTAGCCACTCCTTC
Cxcl10	CCAAGTGCTGCCGTCATTTTC	GGCTCGCAGGGATGATTTCAA
Ccl5	GCTGCTTTGCCTACCTCTCC	TCGAGTGACAAACACGACTGC
Actb	GAGACCTTCAACACCCCAGC	ATGTCACGCACGATTTCCC
Tert	CTAGCTCATGTGTCAAGACCCTCTT	GCCAGCACGTTTCTCTCGTT
POLg	GATGAATGGGCCTACCTTGA	TGGGGTCCTGTTTCTACAGC
RPA1	GGGGATACAAACATAAAGCCCA	CGATAACGCGGCGGACTATT
GAPDH	ACCCACTCCTCCACCTTTGA	CTGTTGCTGTAGCCAAATTCGT
